# Phase 2 study of NAB-paclitaxel in SensiTivE and refractory relapsed small cell lung cancer (SCLC) (NABSTER TRIAL)

**DOI:** 10.1038/s41416-020-0845-3

**Published:** 2020-04-29

**Authors:** Francesco Gelsomino, Marcello Tiseo, Fausto Barbieri, Ferdinando Riccardi, Luigi Cavanna, Antonio Frassoldati, Angelo Delmonte, Lucia Longo, Claudio Dazzi, Saverio Cinieri, Ida Colantonio, Francesca Sperandi, Giuseppe Lamberti, Stefano Brocchi, Lorenzo Tofani, Luca Boni, Andrea Ardizzoni

**Affiliations:** 1 Medical Oncology Unit, Policlinico Sant’Orsola-Malpighi, Bologna, Italy; 2 Medical Oncology Unit, Azienda Ospedaliero-Universitaria of Parma, Parma, Italy; 3 Medical Oncology Unit, Policlinico of Modena, Modena, Italy; 4 Medical Oncology Unit, Azienda Ospedaliera Cardarelli, Napoli, Italy; 5 Medical Oncology Unit, AUSL of Piacenza, Piacenza, Italy; 6 Medical Oncology Unit, Azienda Ospedaliero-Universitaria of Ferrara, Ferrara, Italy; 7 Department of Medical Oncology, Istituto Scientifico Romagnolo per lo Studio e la Cura dei Tumori (IRST) IRCCS, Meldola, Italy; 8 Medical Oncology Unit, AUSL of Modena, Hospital of Carpi, Carpi, Italy; 9 Medical Oncology Unit, AUSL of Romagna, Hospital of Ravenna, Ravenna, Italy; 10 Medical Oncology Unit, Hospital of Brindisi, Brindisi, Italy; 11 Medical Oncology Unit, Hospital of Cuneo, Cuneo, Italy; 12 Radiology Unit, Policlinico Sant’Orsola-Malpighi, Bologna, Italy; 13 Clinical Trial Center, Istituto Toscano Tumori, Azienda Ospedaliero-Universitaria Careggi, Firenze, Italy

**Keywords:** Small-cell lung cancer, Chemotherapy

## Abstract

**Background:**

Despite sensitivity to first-line chemotherapy, most small-cell lung cancer (SCLC) patients relapse. In this setting, topotecan demonstrated modest activity with significant toxicity. Paclitaxel was also active. This study was designed to evaluate activity and safety of nab-paclitaxel in relapsed SCLC.

**Methods:**

In this multicentre prospective Phase 2 trial, patients with refractory or sensitive SCLC progressed to first-line platinum-based chemotherapy received nab-paclitaxel 100 mg/smq on days 1, 8, 15 every 4 weeks up to six cycles, progressive disease or intolerable toxicity. Primary endpoint was investigator-assessed objective tumour response. Secondary endpoints were toxicity, progression-free survival (PFS) and overall survival (OS).

**Results:**

Of the 68 patients treated, partial response was 8% in the refractory cohort and 14% in the sensitive cohort. Most common toxicities of any grade were fatigue (54%), anaemia (38%), neutropenia (29%), leukopenia (26%) and diarrhoea (21%). Median PFS was similar in both refractory (1.8 months) and sensitive cohorts (1.9 months), while median OS was longer in sensitive one (6.6 versus 3.6 months).

**Conclusions:**

Although nab-paclitaxel has shown some modest anti-tumour activity in relapsed SCLC, associated with a favourable toxicity profile, the primary end-point of the study was not met.

**Clinical Trial registration:**

Clinical Trial registration number is ClinicalTrials.gov Identifier: NCT03219762.

## Background

Small cell lung cancer (SCLC) is one of the most aggressive tumours and accounts for ~13–15% of all lung cancers.^1^ Most patients with SCLC have extensive-disease (ED-SCLC) at the time of diagnosis, with a median overall survival (OS) of 8–12 months.^2^

In the last 30 years, platinum-based chemotherapy has been the standard of care in first-line setting, providing an objective response rate (ORR) of 70–80%. Unfortunately, despite high sensitivity to first-line chemotherapy, most SCLC patients eventually develop disease progression.^3^ At relapse, efficacy of second-line treatment is modest and highly influenced by the type and duration of response to prior chemotherapy.^4^ Topotecan, the only approved and marketed drug in Europe specifically for the treatment of relapsed SCLC, showed anti-tumour activity (7% and 21.7%)^5,6^ and a significant improvement in overall survival (OS) over best supportive care (25.9 weeks versus 13.9 weeks, *p* = 0.0104).^5,6^ Nevertheless, it had similar activity (24.3% versus 18.3%) and efficacy (median OS: 25.0 weeks versus 24.7 weeks) to CAV combination chemotherapy.^7^

However, the anti-tumour activity of topotecan is modest and transient and its use is outweighed by its poor compliance and inconvenient schedule.^8^ Therefore, there is a clinical need for more effective and better tolerated treatments.

Paclitaxel has also shown activity in the treatment of SCLC, both alone and in combination with carboplatin, even in refractory relapsed disease.^9–11^ Notably, the use of paclitaxel is encumbered with a significant risk of severe hypersensitivity reactions and cumulative peripheral neurotoxicity that can limit its use.

Nanoparticles Albumin-Bound (Nab)-paclitaxel (Abraxane®; Celgene, Summit, New Jersey) is a new solvent-free formulation of paclitaxel made through high-pressure homogenisation of paclitaxel in presence of serum albumin. In comparison to solvent-based paclitaxel, this formulation, demonstrating a better tumour penetration in preclinical studies, allows reductions in reconstitution volume, infusion time, risk of hypersensitivity reactions, incidence of neutropenia and time needed to recover from peripheral neuropathy.^12–14^

Nab-paclitaxel is currently approved both as single-agent, for the treatment of metastatic breast cancer,^15^ and as combined therapy with gemcitabine or carboplatin in first-line setting, for the treatment of advanced pancreatic adenocarcinoma^16^ or advanced non-small cell lung cancer (NSCLC),^17^ respectively. Three Asian retrospective analyses conducted in relapsed SCLC patients showed some anti-cancer activity of nab-paclitaxel.^18–20^ Since nab-paclitaxel has not been prospectively studied in relapsed SCLC yet, we designed this open-label, prospective Phase 2 trial with the aim to assess its activity and safety in patients with both refractory and sensitive disease.

## Methods

### Study design and participants

Nabster was a prospective, open-label, multicentre, Phase 2 trial evaluating the activity and safety of nab-paclitaxel in SCLC patients who relapsed during or after first-line platinum-based chemotherapy. Patients were prospectively classified according to treatment free interval (TFI), i.e. the interval from the last chemotherapy administration during first-line chemotherapy and the occurrence of progressive disease, as refractory (TFI < 60 days) or *sensitive* (TFI ≥ 60 days).^4^

Patients aged 18 years or older were eligible for study participation if they had a histological or cytological confirmed diagnosis of SCLC, large cell neuroendocrine carcinoma (LCNEC) or undifferentiated neuroendocrine carcinoma of the lung, according to World Health Organization (WHO) classification 2015,^21^ adequate liver, renal and bone marrow functions, measurable disease per Response Evaluation Criteria in Solid Tumors (RECIST) v1.1,^22^ documented radiological evidence of disease progression during or after platinum/etoposide chemotherapy, Eastern Cooperative Oncology Group (ECOG) performance status (PS) 0 to 1. In addition, patients with treated, asymptomatic and stable brain metastases were allowed to be enrolled into the study.

The study protocol was approved by each local institutional ethics committee and conducted in accordance with the ICH Harmonized Tripartite Guidelines for Good Clinical Practice and the Declaration of Helsinki. Written informed consent was obtained from all participants.

The study was sponsored by Gruppo Oncologico Italiano di Ricerca Clinica (GOIRC) and partially supported by Celgene that provided investigational medicinal product and a restricted grant for the management of study procedures. The trial was registered at ClinicalTrials.gov (number NCT03219762) and assigned its Eudract number (2016-000408-27).

### Procedures

Eligible patients received weekly intravenous administration of nab-paclitaxel 100 mg/smq on days 1, 8, 15 of a 28-days cycle until a maximum of six cycles, progressive disease or unacceptable toxicity. Treatment could be continued beyond the 6th cycle in patients with confirmed and prolonged objective response, clinical benefit and good tolerance to study drug. Dose reductions and delays were permitted as per-protocol definitions (Study protocol is available in S.1, Supplemental Data). At screening, disease assessment included a computed tomography (CT) scan of the thorax and upper and lower abdomen with contrast. A brain CT or magnetic resonance imaging (MRI) scan had to be performed only if previously abnormal or clinically indicated.

Tumour response was assessed with computed tomography (CT) scan every 8 weeks (±7 days), according to RECIST criteria v.1.1, and at least 4 weeks after the first observation of a complete or partial response. Furthermore, brain CT scans had to be repeated if initially abnormal or to be performed if clinically indicated. Patients who discontinued nab-paclitaxel without evidence of progressive disease, continued to be evaluated for disease status every 8 weeks, unless they started new anti-cancer therapy. Complete response (CR) was defined as the complete disappearance of all target lesions and all non-target lesions, if present. Partial response (PR) was defined as at least a 30% decrease in the sum of diameters of target lesions, taking as reference the baseline sum diameters. Progressive disease (PD) was defined as at least a 20% increase in the sum of diameters of target lesions, taking as reference the smallest sum on study. The appearance of one or more new lesions and/or unequivocal progression of pre-existing non-target lesions were also considered criteria defining disease progression. Laboratory testing was performed before each study drug administration.

### Outcomes

The primary endpoint was objective tumour response. Tumour response was evaluated according to standard RECIST v.1.1 and based on Investigator’s assessment. Data were reported as percentage of CR, PR, stable disease (SD) and PD. Patients with no tumour assessment after baseline were classified as non-responders. Furthermore, to ensure consistency of tumour response measurements among Centres, CT scans performed for all evaluable patients at baseline and during study treatment could be reviewed by a blinded independent radiological committee (BIRC).

Secondary endpoints were toxicity, progression-free survival (PFS) and overall survival (OS). The assessment of safety was based mainly on the frequency of adverse events; toxicity was measured according to NCI Common Toxicity Criteria Adverse Events (NCI-CTCAE), version 4.03.

PFS was defined as the time from the date of patient’s registration to the date of the evidence of progressive disease, death due to any cause, or the last date the patient was known to be progression-free or alive. OS was calculated from the date of patient’s registration to the date of death from any cause or the last date the patient was known to be alive.

### Statistical design

The aim of this study was to evaluate if nab-paclitaxel objective tumour response rate in each of the two cohorts, sensitive and refractory relapsed SCLC, was sufficient to justify further investigation of the drug in these patients.

In refractory disease, an objective response rate (ORR) ≤ 5% would not have been considered of further interest. According to the Fleming’s single stage design, based on our hypothesis that experimental treatment could guarantee an ORR ≥ 20% (for a 5% significance level and 80% power), 22 patients with *refractory* disease were to be enrolled into the study. An ORR > 5% was considered possible if at least 4 objective responses had been observed.

In *sensitive* disease, an ORR ≤ 15% would not have been considered of further interest. According to the Fleming’s single stage design, based on our hypothesis that experimental treatment could guarantee an ORR ≥ 30% (for a 5% significance level and 80% power), 43 patients with sensitive disease were to be enrolled into the study. An ORR > 15% was considered possible if at least 11 objective responses had been observed.

The study was not designed to perform any comparison between the two cohorts.

Registered population included all patients who were enrolled into the trial. All enrolled patients who received at least one dose of nab-paclitaxel were included in the modified intention-to-treat (mITT) population and considered evaluable for activity and safety.

Descriptive tables were produced for the ORR and the best overall response. Exact binomial method was used to estimate the ORR and its 90% confidence interval.

The assessment of safety was based on the frequency of adverse events that were described as the number (and percentage) of patients reporting any adverse event, as adverse event in each body system and each individual adverse event.

Probabilities of PFS and OS were calculated according to the Kaplan–Meier product-limit method. The data cut-off for analysis was 18 October 2018.

## Results

### Patient and treatment characteristics

**Fig. 1:**
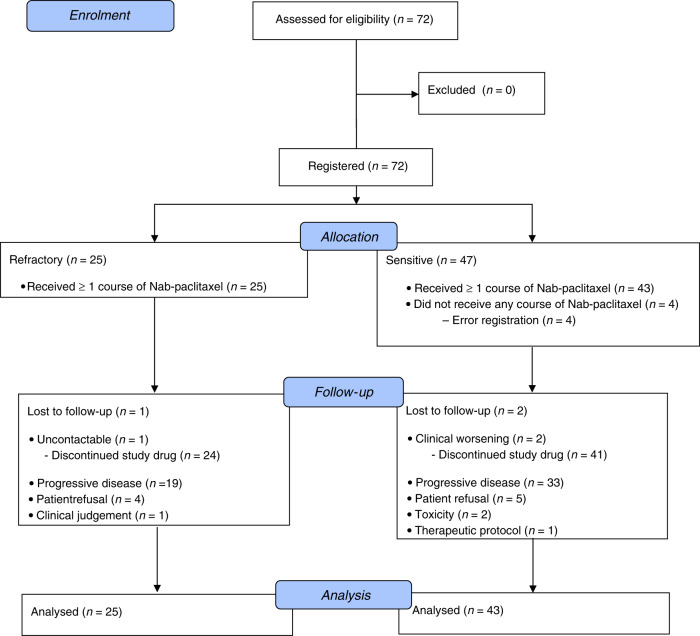
CONSORT flow diagram. It displays the progress of all participants through the NABSTER trial.

Between February 2017 and March 2018, 72 patients were enrolled into the trial from 18 Italian Centres (a list of all participating Centres is available in S2, Supplemental Data). Of them, 68 patients (25 refractory and 43 sensitive) were evaluable for safety and activity and included in the mITT population (Fig. 1). Baseline patients’ characteristics are shown in Table 1. With a median age of 68.5 years (44–80), a male predominance (65%) and a high prevalence of extensive disease (84%), our study population was quite representative of clinical practice. Notably, among patients with extensive disease, 42% had liver involvement, 12% had central nervous system (CNS) disease and 16% had both liver and brain metastases at the time of study enrolment.

**Table 1:** Patients’ demographic characteristics and tumour features.

	Refractory (*n* = 25)	Sensitive (*n* = 43)
Age, median in years (range)	65 (52–80)	69 (44–79)
Gender		
Female	11 (44.0%)	13 (30.2%)
Male	14 (56.0%)	30 (69.8%)
ECOG performance status (PS)		
0	8 (32.0%)	22 (51.2%)
1	17 (68.0%)	21 (48.8%)
Histology		
SCLC	22 (88.0%)	37 (86.0%)
LCNEC	2 (8.0%)	3 (7.0%)
NEC undifferentiated	1 (4.0%)	3 (7.0%)
Tumor stage		
LD	5 (20.0%)	6 (14.0%)
ED	20 (80.0%)	37 (86.0%)
Liver	10 (50.0%)	14 (37.8%)
Brain	4 (20.0%)	3 (8.1%)
Liver + Brain	3 (15.0%)	6 (16.2%)
Treatment free interval, median in days (range)	20 (0–57)	123 (61–820)
Prior treatment		
Chemotherapy	24 (96.0%)	43 (100%)
Chemotherapy + ICI	1 (4.0%)	0
Type of chemotherapy		
Cisplatin + Etoposide	4 (16.0%)	13 (30.2%)
Carboplatin + Etoposide	21 (84.0%)	30 (69.8%)
Prior radiotherapy		
No	19 (76.0%)	13 (30.2%)
Yes	6 (24.0%)	30 (69.8%)

*SCLC* small cell lung cancer, *LCNEC* large cell neuroendocrine carcinoma, *LD* limited disease, *ED* extensive disease, *ICI* immune checkpoint inhibitor.

The mean number of courses per patient was 2.48 in refractory group and 3.00 in sensitive one. Only 12% of patients concluded the planned treatment courses. Dose reduction occurred 61 times (32%) in 39 patients, mainly due to haematological toxicity (26 cases). Dose delay was reported 49 times (25%) in 33 patients. Despite dose reductions and delays, the relative dose intensity remained good (76% in *refractory* cohort and 80% in sensitive cohort). All information on treatment distribution is available in S.3 (Supplemental data).

### Tumour response

According to Investigator’s assessment, PR was observed in 2 (8%; IC 90%, 1.7–24.0) patients in refractory cohort and in 6 (13.9%; IC 90%, 6.6–26.1) patients in sensitive one. Thirteen (19.1%) patients had SD, 5 patients (20.0%) of them in refractory cohort, while 36 (52.9%) patients had PD as best response, of whom 14 (56.0%) in refractory group (Table 2).

**Table 2:** Best overall response based on both Investigator and BIRC assessment.

	Investigator’s assessment			BIRC’s assessment		
	Refractory (*n*. 25)	Sensitive (*n*. 43)	Total	Refractory (*n*. 25)	Sensitive (*n*. 43)	Total
CR	0	0	0	0	0	0
PR	2 (8.0)	6 (13.9)	8 (11.8)	4 (16.0)	8 (18.6)	12 (17.6)
SD	5 (20.0)	8 (18.6)	13 (19.1)	4 (16.0)	7 (16.3)	11 (16.2)
PD	14 (56.0)	22 (51.2)	36 (52.9)	13 (52.0)	21 (41.8)	34 (50.0)
NE	4 (16.0)	7 (16.3)	11 (16.2)	4 (16.0)	7 (16.3)	11 (16.2)
Total	25 (100)	43 (100)	68 (100)	25 (100)	43 (100)	68 (100)

*CR* complete response, *PR* partial response, *SD* stable disease, *PD* progressive disease, *NE* not evaluated, *BIRC* blinded independent radiological committee.

**Fig. 2:**
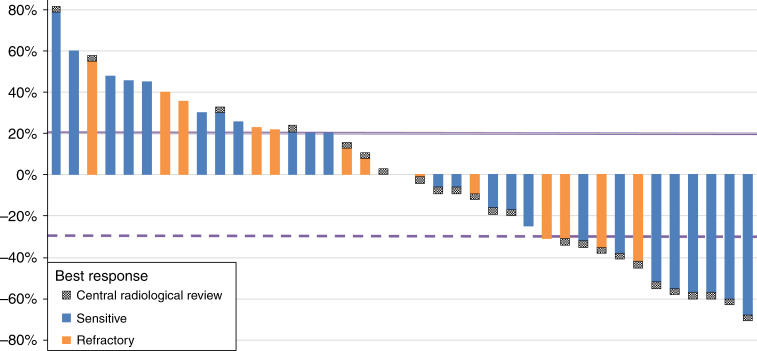
Waterfall plot. It describes the changes in tumor size in all evaluable participants with target lesions. Blue bars represent sensitive patients, while orange bars are refractory ones. The black/white signal at the top of each bar corresponds to central radiological review for each individual patient.

Investigator-assessed responses were reviewed by a BIRC. According to central review assessment (Table 2), PR was observed in 4 (16.0%; IC 90%, 6.1–33.5) patients in refractory cohort and in 8 (18.6%; IC 90%, 9.9–31.4) patients in sensitive one. Eleven (16.2%) had SD, 4 patients (16.0%) of them in refractory cohort, while 34 (50.0%) patients had PD as best response, of whom 13 (52.0%) in refractory group. Finally, 11 (16.2%) patients was not evaluated for response, 4 in refractory group and 7 in sensitive one. Waterfall plot (Fig. 2) shows the distribution and depth of response in patients evaluated for target lesions.

Notably, among 16 (28%) patients with CNS involvement at baseline, 5 (31.2%) patients obtained a brain disease control, including also 2 (22.2%) patients with concomitant CNS and liver disease.

### Safety

All 68 patients included in the mITT population were evaluable for safety. Adverse events of any grade occurred in 53 patients (77.9%) (Table 3). Haematological and non-haematological toxicities of any grade were reported in 36 (52.9%) and in 49 (72.0%) patients, respectively, whereas the same toxicities of grade 3–4 were observed in 9 (13.2%) and 6 (8.8%) patients, respectively. The most frequent adverse event of any grade was fatigue (54.4%), the only toxicity which led to permanent discontinuation of study drug in 2 (4.6%) patients. Only one treatment-related adverse event of grade 4 (leuko-neutropenia) was reported throughout the study period. There was no treatment-related death.

**Table 3:** Toxicity profile.

	Any grade	Grade ≥ 3
Fatigue	37 (54.4%)	3 (4.4%)
Anemia	25 (36.7%)	1 (1.4%)
Neutropenia	20 (29.4%)	7 (10.3%)
Leukopenia	18 (26.4%)	3 (4.4%)
Diarrhea	14 (20.5%)	0
Nausea	13 (19.1%)	0
Peripheral neuropathy	13 (19.1%)	0
Fever without neutropenia	10 (14.7%)	0
Vomiting	8 (11.7%)	1 (1.4%)
Trombocytopenia	7 (10.3%)	0
Constipation	6 (8.8%)	0
Skin toxicity	5 (7.3%)	1 (1.4%)
Mucositis	5 (7.3%)	0
Liver toxicity	3 (4.4%)	1 (1.4%)
Renal toxicity	1 (1.4%)	0

### Survival

**Fig. 3:**
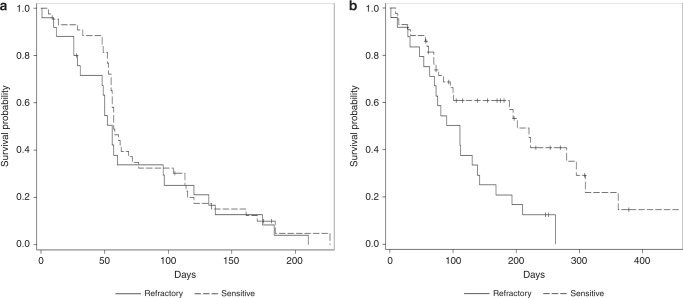
PFS and OS in modified ITT population. Probabilities of PFS (**a**) and OS (**b**) were calculated according to the Kaplan–Meier product-limit method. In both graphs (**a** and **b**), continuous and dashed curves represent survival probabilities in refractory and sensitive cohorts, respectively.

The median duration of follow-up was 8.4 months (IQR, interquartile range: 5.8–12.4). Median PFS (mPFS) was 1.84 months (IC 95%, 1.02–3.16) in *refractory* cohort, and 4.2% (IC 95%, 0.3–17.7) of these patients were free from disease progression at 6 months (Fig. 3a). Similar results were observed in *sensitive* group, for which mPFS was 1.88 months (IC 95%, 1.81–2.37), with a 6-month PFS rate of 10.1% (IC 95%, 3.2–21.5) (Fig. 3a).

Median OS (mOS) was 3.65 months (IC 95%, 2.07–4.57) in refractory cohort and 20.9% (IC 95%, 7.6–38.6) of these patients were alive at 6 months (Fig. 3b), whereas in sensitive cohort mOS was 6.64 months (IC 95%, 3.16–9.70), with a 6-month OS rate of 60.8% (IC 95%, 44.1–73.9) (Fig. 3b). At the time of data cut-off, no patient was still being treated, although 4 (5.9%) patients (1 in refractory cohort and 3 patients in sensitive one) had no event, and 58 (85.3%) patients progressed, of whom 22 (88.0%) were refractory. Twenty-one (30.9%) patients were alive (3 refractory and 18 sensitive), while 47 (69.1%) patients were dead, 40 (58.8%) of them due to disease progression (19 and 21 patients in refractory and sensitive cohorts, respectively).

## Discussion

Based on its poor prognosis and survival plateau achieved in the last decades, SCLC has been defined one of the recalcitrant cancers. Till now, several treatment strategies and clinical trial designs have been developed with daunting results. Therefore, there is an urgent need for additional and effective therapeutic innovations. The impressive results of immune checkpoint inhibitors, such as the monoclonal antibodies directed against programmed cell death-1 (PD-1) and its ligand (PD-L1), for the treatment of different solid tumours, have led to evaluate them also in SCLC. In the last year, two randomised, controlled Phase 3 trials showed that adding atezolizumab (IMpower-133 study) or durvalumab (CASPIAN study), two antibodies directed against PD-L1, to standard first-line chemotherapy led to a statistically significant improvement in terms of OS in patients with ED-SCLC, although this benefit would not be considered clinically significant.^23,24^

In second-line setting, different clinical studies are investigating the efficacy of several new agents, either alone or combined to standard chemotherapy.

Our study is the first prospective trial of nab-paclitaxel for relapsed SCLC. Overall, this trial showed a modest anti-cancer activity, so that it did not meet its primary endpoint (ORR), in both refractory and sensitive cohorts. Based on investigator’s assessment and study design, there were 2 tumour responses (ORR, 8%) out of 4 or more required in refractory group and 6 tumour responses (ORR, 13.9%) out of 11 or more required in sensitive one, needed to reach the primary objective of the study. However, after central independent radiological review, two additional cases of objective response were identified in the refractory group which would qualify the study as positive, at least in this cohort. Secondary endpoints of the study included PFS, OS and toxicity. Data on survival outcomes confirmed the dismal prognosis of these patients, with a mPFS less than 2 months in both refractory and sensitive cohorts and a mOS that was almost double in sensitive group (6.64 months) compared to refractory one (3.65 months). Furthermore, although nearly 30% of patients with CNS involvement at baseline had a brain disease control, 11 (68.7%) out of 16 patients experienced a rapid progressive disease (within 1–2 courses), including two patients with early death. These data confirmed the unfavourable prognostic role of CNS involvement, especially in relapsed SCLC.

Our results were similar to those reported from a retrospective study^18^ in which 9 of the 14 enrolled patients were treated with nab-paclitaxel, as third-line or later. In this subgroup, ORR, mPFS and mOS were 11%, 2.0 months and 4.0 months, respectively. Almost all patients were refractory to first-line chemotherapy regimen, but the authors did not report any information on the prevalence of brain and liver metastases in this population.

Similarly, a retrospective analysis reported outcome of 31 heavily pre-treated Japanese SCLC patients of whom only 4 received nab-paclitaxel, preventing any meaningful consideration on the efficacy of this agent.^20^

In our study, the discordance in terms of ORR between local and central assessment has been mainly due, at least in some cases, to an improper application of RECIST v1.1 by local radiologists. For example, two refractory patients considered as stable were reclassified as responders after central radiological review because of a misleading interpretation of two target liver lesions in one case and two pathological mediastinal lymph nodes in the other one. These results in refractory cohort are not similar to those reported from different Phase 2 trials that showed how paclitaxel had a promising anti-tumour activity, reaching a response rate of 20–29%.^9,25^ A higher response rate (41%) was reported from a Phase 2 trial of irinotecan administered in 30 Japanese patients with relapsed SCLC. However, it is reasonable to believe that patient population included into this study was “positively” selected. In fact, all patients had ECOG PS 0 or 1, one third of them had LD-stage, 60% had sensitive recurrent disease, with only 10% and 13% of patients having brain and liver involvement, respectively.^26^ Similar results were reported from a multicentre, single-arm Phase 2 basket study of lurbinectedin, a RNA polymerase II inhibitor, in patients across advanced solid tumours. Thirty-seven (35.2%) out of 105 enrolled SCLC patients had a partial response. Overall, median PFS and OS times were 3.9 months (95% CI, 2.6–4.6) and 9.3 months (95% CI, 6.3–11.8). According to TFI (<or ≥90 days), these clinical outcomes have more than doubled in sensitive patients (45%, 4.6 months and 11.9 months) compared to refractory ones (22.2%, 2.6 months and 5.0 months). Lurbinectedin showed a favourable and manageable toxicity profile. The most common grade 1–2 adverse events were fatigue (51.4%), nausea (32.4%), decreased appetite (21%), vomiting (18.1%) and diarrhoea (12.4%). Grade 3–4 adverse events included neutropenia (22.9%), anaemia and fatigue (6.7% each), febrile neutropenia and thrombocytopenia (4.8% each).^27^

To date, topotecan remains the only drug approved for relapsed SCLC patients, based on the results of different Phase 2–3 trials that showed a response rate of 7–38% among sensitive patients and of 2–7% among refractory ones.^5–7,28,29^ A recent meta-analysis described clinical outcomes of 1347 SCLC patients treated with topotecan from 14 prospective trials.^30^ Objective tumour response and 6-month OS rates were 5% and 37% in refractory patients and 17% and 57% in sensitive ones, respectively. Notably, these data are in line with the results of our study. Results from clinical studies investigating the role of anti-PD-1/PD-L1 drugs in second- or further-line setting were conflicting so far, particularly when used as single-agent.^31–36^ In the recently reported Phase 3 CheckMate-331 trial of nivolumab, a human IgG4 monoclonal antibody against PD-1, 569 SCLC patients relapsed on or following platinum-based chemotherapy were randomised (1:1) to receive either nivolumab (*N* = 284) or standard second-line chemotherapy (topotecan or amrubicin, *N* = 285).^37^ Results of this study showed that, after 7.0–7.6 months of median follow-up, nivolumab did not yield a significant survival improvement (primary endpoint) compared to the standard chemotherapy arm (7.5 months [95% CI 5.6–9.2] versus 8.4 months [95% CI 7.0–10.0], *p* = 0.11). This confirms that, at least in a subset of relapsed SCLC patients, chemotherapy is the option of choice.

Based on safety, nab-paclitaxel has shown a favourable toxicity profile, particularly considering historical data on topotecan. Nab-paclitaxel was well tolerated, and the proposed schedule was feasible. The most common grade 3–4 adverse events were neutropenia (10%), leukopenia and fatigue (4% each) and anaemia (1%). Conversely, topotecan was encumbered with a high incidence of severe (grade 3–4) haematological toxicity, including neutropenia (69%), thrombocytopenia (41%) and anaemia (24%).^30^

Although nab-paclitaxel has demonstrated a not negligible anti-tumour activity, particularly in refractory relapsed SCLC, associated with a favourable toxicity profile, the primary end-point of the study was not meet. Based on these results, we believe that further studies comparing nab-paclitaxel to the current standard-of-care topotecan would not be justified.

## Supplementary information

Supplemental Data

## Data Availability

Anonymised dataset may be available from the corresponding author on reasonable request.
