# Correction to: Phase 2 study of NAB-paclitaxel in SensiTivE and refractory relapsed small cell lung cancer (SCLC) (NABSTER TRIAL)

**DOI:** 10.1038/s41416-021-01439-1

**Published:** 2021-05-19

**Authors:** Francesco Gelsomino, Marcello Tiseo, Fausto Barbieri, Ferdinando Riccardi, Luigi Cavanna, Antonio Frassoldati, Angelo Delmonte, Lucia Longo, Claudio Dazzi, Saverio Cinieri, Ida Colantonio, Francesca Sperandi, Giuseppe Lamberti, Stefano Brocchi, Lorenzo Tofani, Luca Boni, Andrea Ardizzoni

**Affiliations:** 1 Medical Oncology Unit, Policlinico Sant’Orsola-Malpighi, Bologna, Italy; 2 Medical Oncology Unit, Azienda Ospedaliero-Universitaria of Parma, Parma, Italy; 3 Medical Oncology Unit, Policlinico of Modena, Modena, Italy; 4 Medical Oncology Unit, Azienda Ospedaliera Cardarelli, Napoli, Italy; 5 Medical Oncology Unit, AUSL of Piacenza, Piacenza, Italy; 6 Medical Oncology Unit, Azienda Ospedaliero-Universitaria of Ferrara, Ferrara, Italy; 7 Department of Medical Oncology, Istituto Scientifico Romagnolo per lo Studio e la Cura dei Tumori (IRST) IRCCS, Meldola, Italy; 8 Medical Oncology Unit, AUSL of Modena, Hospital of Carpi, Carpi, Italy; 9 Medical Oncology Unit, AUSL of Romagna, Hospital of Ravenna, Ravenna, Italy; 10 Medical Oncology Unit, Hospital of Brindisi, Brindisi, Italy; 11 Medical Oncology Unit, Hospital of Cuneo, Cuneo, Italy; 12 Radiology Unit, Policlinico Sant’Orsola-Malpighi, Bologna, Italy; 13 Clinical Trial Center, Istituto Toscano Tumori, Azienda Ospedaliero-Universitaria Careggi, Firenze, Italy

**Keywords:** Small-cell lung cancer, Chemotherapy

Correction to: *British Journal of Cancer* 10.1038/s41416-020-0845-3, published online 29 April 2020

The original version of this article unfortunately contained a mistake in the affiliation of Dr. Delmonte. The correct affiliation should be: Department of Medical Oncology, Istituto Scientifico Romagnolo per lo Studio e la Cura dei Tumori (IRST) IRCCS, Meldola, Italy. The original article has been corrected.

