# Supplementary material for: Phase 2 study of NAB-paclitaxel in SensiTivE and refractory relapsed small cell lung cancer (SCLC) (NABSTER TRIAL)
Source: Br J Cancer. 2020 Apr 29;123(1):26–32. doi: 10.1038/s41416-020-0845-3 (PMC7341887; doi:10.1038/s41416-020-0845-3)
Supplement: Supplementary file 1 — Supplemental Data [file 41416_2020_845_MOESM1_ESM.doc]

**Supplementary information**

**S1.** Nabster full protocol

**S2.** List of all participating Centres

**S3.** Treatment distribution

**S4.** List of the Ethics Committees that approved the study

**S.1.**

**Phase II study of NAB-paclitaxel in *SensiTivE* and *Refractory* relapsed SCLC (NABSTER trial)**

Protocol code: GOIRC-02-2016

Eudract Number: 2016-000408-27

Sponsor: Gruppo Oncologico Italiano di Ricerca Clinica (GOIRC)

Study Coordinators: Andrea Ardizzoni, Marcello Tiseo

Steering Committee: Andrea Ardizzoni, Marcello Tiseo, Michele Tognetto, Luca Boni, Francesco Gelsomino

Coordinator Site: UOC di Oncologia Medica, Azienda Ospedaliero-Universitaria Sant’Orsola Malpighi, Bologna

Protocol Version: 1.0 of 15.01.2016

**Confidentiality Statement**

The information contained in this document, is the property of GOIRC, and therefore provided to you in confidence as an investigator, potential investigator or consultant, for review by you, your staff and an applicable Independent Ethics Committee. This information will not be disclosed to others without written authorization.

**STUDY STAFF**

**PRINCIPAL INVESTIGATOR**

*Dr. Andrea Ardizzoni*

Director of Medical Oncology Unit,

Dept. Oncology-Haematology,

S. Orsola-Malpighi University Hospital

Via Albertoni 15, 40138 Bologna, Italy

Phone/Fax: +390512142206/+390516362508

E-mail: [andrea.ardizzoni@aosp.bo.it](mailto:andrea.ardizzoni@aosp.bo.it)

**SCIENTIFIC STUDY COORDINATOR**

*Dr. Marcello Tiseo*

Medical Oncology Unit

University Hospital of Parma

Via Gramsci 14, 43126, Parma, Italy

Phone/Fax: +390521702316/+390521995448

e-mail: mtiseo@ao.pr.it

**SCIENTIFIC SECRETARIAT**

*Dr. Francesco Gelsomino*

Medical Oncology Unit,

Dept. Oncology-Haematology,

S. Orsola-Malpighi University Hospital

Via Albertoni 15, 40138 Bologna, Italy

Phone/Fax: +390512142204/+390516362508

E-mail: [francesco.gelsomino@aosp.bo.it](mailto:andrea.ardizzoni@aosp.bo.it)

**CLINICAL TRIAL OFFICE**

*Dr. Michele Tognetto*

Medical Oncology Unit,

Dept. Oncology-Haematology,

S. Orsola-Malpighi University Hospital

Via Albertoni 15, 40138 Bologna, Italy

Phone/Fax: +390512142204/+390516362508

E-mail: [michele.tognetto@aosp.bo.it](mailto:michele.tognetto@aosp.bo.it); [nabsterstudy@gmail.com](mailto:nabsterstudy@gmail.com)

**STATISTICIAN**

*Dr. Luca Boni*

Istituto Toscano Tumori

c/o AOU Careggi – Cubo 3 – Edificio 27B

V.le G. Pieraccini, 6 – 50139 Firenze

Phone: +39.055.794.4582

E-mail: luca.boni@ittumori.it

**AMENDMENTS**

| **NUMBER** | **VERSION** | **DESCRIPTION** | **APPROVAL** |
| --- | --- | --- | --- |
|  |  |  |  |

**INVESTIGATOR AGREEMENT**

I have read the Protocol entitled *“Phase II study of Nab-paclitaxel in sensitive and refractory relapsed SCLC (NABSTER trial)”* and I agree to conduct the study as detailed herein and in compliance with ICH Guidelines for good clinical Practice and applicable regulatory requirements.

Principal Investigator

Dr. Andrea Ardizzoni

Signature ______________________________

Dr. ____________________________________

Signature _______________________________

**TABLE OF CONTENT**

PROTOCOL SYNOPSIS [9](#__RefHeading___Toc314430062)

LIST OF ABBREVIATIONS [13](#__RefHeading___Toc314430063)

1. BACKGROUND AND INTRODUCTION [14](#__RefHeading___Toc314430064)

1.1 Background  [14](#__RefHeading___Toc314430065)

1.2 Nab-paclitaxel (Abraxane®) [14](#__RefHeading___Toc314430066)

*1.2.1 Introduction* [**14**](#__RefHeading___Toc314430067)

*1.2.2 Preclinical experience* [**14**](#__RefHeading___Toc314430068)

*1.2.3 Phase I - II trials* [**15**](#__RefHeading___Toc314430069)

*1.2.4 Pharmacokinetics* [**15**](#__RefHeading___Toc314430070)

*1.2.5 Phase III trials* [**16**](#__RefHeading___Toc314430071)

*1.2.6 Safety profile* [**17**](#__RefHeading___Toc314430072)

1.3 Rationale of the study [19](#__RefHeading___Toc314430073)

2. TRIAL DESIGN [21](#__RefHeading___Toc314430074)

3. OBJECTIVES AND END-POINTS [22](#__RefHeading___Toc314430075)

3.1 Primary objective [22](#__RefHeading___Toc314430076)

3.2 Secondary objectives [22](#__RefHeading___Toc314430077)

3.3 Primary End-point [22](#__RefHeading___Toc314430078)

3.4 Secondary End-points [22](#__RefHeading___Toc314430079)

4. STUDY POPULATION [23](#__RefHeading___Toc314430080)

4.1 Inclusion criteria [23](#__RefHeading___Toc314430081)

4.2 Exclusion criteria [24](#__RefHeading___Toc314430082)

5. DRUG AND SCHEDULE, TOXICITY AND DOSE MODIFICATIONS [25](#__RefHeading___Toc314430083)

5.1 Nab-paclitaxel (Abraxane®): drug, initial dose and schedule   [25](#__RefHeading___Toc314430084)

5.2 Treatment duration   [26](#__RefHeading___Toc314430085)

5.3 Withdrawal criteria   [26](#__RefHeading___Toc314430086)

5.4 Dose and schedule modifications   [27](#__RefHeading___Toc314430087)

*5.4.1 Day 1 of each cycle (from second cycle and beyond)* [**27**](#__RefHeading___Toc314430088)

*5.4.2 Day 8, 15 of each cycle* [**27**](#__RefHeading___Toc314430089)

6. CONCOMITANT TREATMENTS [30](#__RefHeading___Toc314430090)

6.1 Drugs with proarrhythmic potential   [30](#__RefHeading___Toc314430091)

6.2 Inhibitors and inducers of CYP3A4 and CYP2C8   [30](#__RefHeading___Toc314430092)

6.3 Anticoagulants   [30](#__RefHeading___Toc314430093)

6.4 Supportive care in case of toxicity or palliative radiotherapy   [30](#__RefHeading___Toc314430094)

6.5 Other concomitant therapies   [30](#__RefHeading___Toc314430095)

7. CLINICAL EVALUATION, LABORATORY TESTS AND FOLLOW-UP [31](#__RefHeading___Toc314430096)

7.1 Before treatment start [31](#__RefHeading___Toc314430097)

7.2 During treatment   [31](#__RefHeading___Toc314430098)

7.3 At the end of treatment   [32](#__RefHeading___Toc314430099)

7.4 After progression of the disease   [32](#__RefHeading___Toc314430100)

7.5 Summary table   [33](#__RefHeading___Toc314430101)

8. CRITERIA OF TUMOR EVALUATION [34](#__RefHeading___Toc314430102)

8.1 Objective tumor response [34](#__RefHeading___Toc314430103)

*8.1.1 Measurability of tumor lesions at baseline* [**34**](#__RefHeading___Toc314430104)

*8.1.2 Tumor response evaluation* [**35**](#__RefHeading___Toc314430105)

*8.1.3 Reporting of results* [**37**](#__RefHeading___Toc314430106)

9. EVALUATION OF SAFETY [37](#__RefHeading___Toc314430107)

9.1 Monitoring, Recording and Reporting of Adverse Events [37](#__RefHeading___Toc314430108)

9.2 General evaluation of side-effects   [38](#__RefHeading___Toc314430109)

9.3 Abnormal Laboratory Values [40](#__RefHeading___Toc314430116)

9.4 Pregnancy [40](#__RefHeading___Toc314430117)

9.5 Expedited Reporting of Adverse Events [40](#__RefHeading___Toc314430119)

*9.5.1 Reporting to Regulatory Authorities and the Ethics Committee* [**40**](#__RefHeading___Toc314430120)

*9.5.2 Immediate reporting by Investigator to Sponsor and Sponsor to Celgene* [**40**](#__RefHeading___Toc314430121)

9.6 Toxic deaths   [41](#__RefHeading___Toc314430122)

9.7 Evaluability of toxicity   [41](#__RefHeading___Toc314430123)

10. STATISTICAL CONSIDERATIONS [41](#__RefHeading___Toc314430124)

10.1 Statistical design [41](#__RefHeading___Toc314430125)

*10.1.1 Sample size* [**41**](#__RefHeading___Toc314430126)

*10.1.2 Stratifications* [**42**](#__RefHeading___Toc314430127)

10.2 Statistical analysis plan [42](#__RefHeading___Toc314430128)

*10.2.1 Primary and secondary endpoints* [**42**](#__RefHeading___Toc314430129)

*10.2.2 Analysis populations’* [**42**](#__RefHeading___Toc314430130)

*10.2.3 Statistical methods* [**42**](#__RefHeading___Toc314430131)

*10.2.4 Prognostic factor analyses*   [**43**](#__RefHeading___Toc314430132)

*10.2.5 Data recording and display* [**43**](#__RefHeading___Toc314430133)

10.3 Interim analyses   [44](#__RefHeading___Toc314430134)

10.4 End of study   [44](#__RefHeading___Toc314430135)

11. DATA MONITORING [44](#__RefHeading___Toc314430136)

12. INVESTIGATOR AUTHORIZATION PROCEDURE [44](#__RefHeading___Toc314430137)

13. PATIENT REGISTRATION PROCEDURE [44](#__RefHeading___Toc314430138)

14. FORMS AND PROCEDURES FOR COLLECTING DATA [45](#__RefHeading___Toc314430139)

15. QUALITY ASSURANCE [45](#__RefHeading___Toc314430140)

15.1 Control of data consistency [45](#__RefHeading___Toc314430141)

15.2 Central review procedures [45](#__RefHeading___Toc314430142)

16. ETHICAL CONSIDERATIONS [45](#__RefHeading___Toc314430143)

16.1 Patient protection [45](#__RefHeading___Toc314430144)

16.2 Subject identification [45](#__RefHeading___Toc314430145)

16.3 Informed consent [46](#__RefHeading___Toc314430146)

17. ADMINISTRATIVE RESPONSABILITIES [46](#__RefHeading___Toc314430147)

17.1 The study coordinators [46](#__RefHeading___Toc314430148)

17.2 Clinical Trial Office and Data Center [46](#__RefHeading___Toc314430149)

18. TRIAL SPONSORSHIP [47](#__RefHeading___Toc314430150)

19. TRIAL INSURANCE [47](#__RefHeading___Toc314430151)

20. PUBLICATION POLICY [47](#__RefHeading___Toc314430152)

21. REFERENCES 49

#

# PROTOCOL SYNOPSIS

| **Study Title** | Phase II study of Nab-paclitaxel in *sensitive* and *refractory* relapsed SCLC (NABSTER trial, GOIRC-02-2016) |
| --- | --- |
| **Sponsor** | Gruppo Oncologico Italiano di Ricerca Clinica (GOIRC) |
| **Objective** | To assess activity of weekly nab-paclitaxel as treatment in patients with *sensitive* or *refractory* SCLC relapsed after cisplatin or carboplatin and etoposide first-line chemotherapy |
| **Study design** | This is an open label, multicentre, phase II study evaluating the activity and safety of Nab-paclitaxel in patients with *sensitive* or *refractory* SCLC who relapsed after cisplatin or carboplatin and etoposide first-line chemotherapy. |
| **Number of Patients** | Total number of patients: 65 SCLC patients (22 *refractory* SCLC patients and 43 *sensitive* SCLC patients), at up to 27 national centers |
| **Inclusion Criteria** | - Pathologically (histology or cytology) confirmed diagnosis of small cell lung cancer (SCLC) or large-cell neuroendocrine carcinoma (LCNEC) or poorly differentiated (G3) neuroendocrine cancer of the lung (according to WHO classification 2015) - Male or female and ≥ 18 years of age - Life expectancy ≥ 12 weeks - Have progressed after or during platinum-based standard chemotherapy regimen (cisplatin or carboplatin and etoposide) for first-line treatment of SCLC, either limited stage (LD) or extensive stage (ED) disease and have not received any other treatment (except for immunotherapy as maintenance treatment), including re-treatment with front-line regimen - Have measurable disease per Response Evaluation Criteria in Solid Tumors, version 1.1 (RECIST 1.1); clear radiological evidence of disease progression after first-line therapy has to be documented; no previous radiotherapy on the only site of measurable or evaluable disease, unless that site had subsequent evidence of progression - Eastern Cooperative Oncology Group (ECOG) performance status (PS) of 0 or 1 - Patients with treated brain metastases with stable lesions for at least 2 weeks and off steroids or on a stable dose of steroids. Radiotherapy must have been completed a minimum of 14 days prior to registration, and patients must have recovered from AEs related to radiotherapy to < grade 1 (except alopecia) - For Females: must be postmenopausal (defined as occurring 12 months after last menstrual period) before the screening visit, or are surgically sterile. If they are of childbearing potential, a negative serum pregnancy test prior to study entry has to be documented; furthermore, they agree to practice 2 effective methods of contraception, at the same time, from the time of signing the informed consent form (ICF) through 30 days after the last dose of study drug, or agree to practice true abstinence, when this is in line with the preferred and usual lifestyle of the subject - For Males: even if surgically sterilized (i.e. post-vasectomy status) agree to practice effective barrier contraception during the entire study treatment period and through 6 months after the last dose of study drug, or practice true abstinence, when this is in line with the preferred and usual lifestyle of the subject - Screening clinical laboratory values as specified below:   - Absolute neutrophil count (ANC) ≥ 1500/mm3, platelet count ≥ 100,000/mm3 and haemoglobin ≥ 9 g/dL   - Total bilirubin < 1.5 the institutional upper limit of normal (ULN)   - Serum alanine aminotransferase (ALT) or aspartate aminotransferase (AST) < 2.5 the institutional ULN (< 5 if liver function test elevations are due to liver metastases)   - Creatinine < 1.5 institutional ULN or estimated creatinine clearance using the Cockcroft-Gault formula ≥ 30 mL/minute for patients with creatinine levels above institutional limits - Stable medical condition, including the absence of acute exacerbations of chronic illnesses, serious infections, or major surgery within 4 weeks before registration, and otherwise noted in other inclusion/exclusion criteria - Recovered (i.e., ≤ Grade 1 toxicity) from effects of prior anticancer therapy, except alopecia - Prior radiotherapy is allowed provided that it has been completed more than 2 weeks before starting Nab-paclitaxel - Ability to comply with protocol requirements - The patient or the patient’s legal representative has to be able to provide written informed consent. Voluntary written consent must be given before performance of any study-related procedure not part of standard medical care, with the understanding that consent may be withdrawn by the patient at any time without prejudice to future medical care |
| **Exclusion criteria** | Patients meeting any of the following exclusion criteria are not eligible to the study:   - Any prior not platinum-based chemotherapy treatment for SCLC or large-cell neuroendocrine carcinoma (LCNEC) (immunotherapy is allowed as maintenance treatment) - Prior treatment with Nab-paclitaxel, paclitaxel or any other taxane agent - Known hypersensitivity to Cremophor EL®, paclitaxel, or its components - Any comorbid condition or unresolved toxicity that would preclude administration of weekly Nab-paclitaxel - Prior history of Grade ≥ 2 neurotoxicity that is not resolved to ≤ Grade 1 - Patients with symptomatic and/or progressive brain metastases or with carcinomatous meningitis - Diagnosed with or treated for another malignancy within 3 years before the first dose of study drug, or previously diagnosed with another malignancy and have any evidence of residual disease. Patients with non-melanoma skin cancer or carcinoma in situ of any type may be enrolled in the study if they have undergone complete resection and no evidence of active disease is present - History of myocardial infarction, unstable symptomatic ischemic heart disease, uncontrolled hypertension despite appropriate medical therapy, any ongoing cardiac arrhythmias of Grade > 2, thromboembolic events (e.g., deep vein thrombosis, pulmonary embolism, or symptomatic cerebrovascular events), or any other cardiac condition (egg, pericardial effusion or restrictive cardiomyopathy) within 6 months before receiving the first dose of study drug. Chronic stable atrial fibrillation on stable anticoagulant therapy is allowed. Patients with a pacemaker may be enrolled in the study upon discussion with the project clinician - Infection requiring IV antibiotic therapy or other serious infection within 14 days before the first dose of study drug - For female subjects: positive serum pregnancy test, pregnancy or breast feeding - Surgery within 3 weeks (or 2 weeks for a minor surgery) before study enrolment and not fully recovered to baseline or to a stable clinical status. Insertion of a vascular device is allowed - Unwilling or unable to comply with the protocol or cooperate fully with the investigator and site personnel |
| **Treatment**  Test product, dose and mode of administration  Duration of treatment | Eligible patients will be registered to receive Nab-paclitaxel (30-min infusion) 100 mg/sqm weekly on days 1, 8, 15 q 28 days. Patients will be reassessed with CT scan every 2 cycles. Chemotherapy will be continued until a maximum of 6 courses or progressive disease or intolerable toxicity or patient refusal. In patients with confirmed and prolonged disease response, clinical benefit and good tolerance to study drug treatment, the investigators can evaluate to continue therapy beyond 6^th^ cycle, after discussion with Principal Investigator (PI) of the study |
| **Primary End-point**  **Secondary end-points** | The primary end-point is objective tumor response that will be evaluated according to standard RECIST 1.1 criteria and will be based on the Investigator’s assessment. Data will be reported as percentage of complete responses (CRs), partial responses (PRs), stable disease (SD) and progressive disease (PD). Exact binomial method will be used to estimate the response rate (CR+PR) and its 95% confidence interval. Patients with no tumor assessment after baseline will be classified as non-responders.  The secondary end-points are:  1) Toxicity: the assessment of safety will be based mainly on the frequency of adverse events; toxicity will be measured according to NCI Common Toxicity Criteria Adverse Event (CTCAE), version 4.03.  2) Progression Free Survival (PFS) will be calculated from the patient registration to the evidence of progressive disease, or death, or the last date the patient was known to be progression-free or alive.  3) Overall Survival (OS) will be calculated from the registration to death from any cause, or the last date the patient was known to be alive. |
| **Statistical methods** | In the study, 2 cohorts of *refractory* and *sensitive* patients will be enrolled.  *Refractory* and *sensitive* disease is defined according to treatment free interval (TFI), i.e. the interval from the last chemotherapy administration during first-line therapy and the occurrence of progressive disease, instrumentally assessed. Disease is considered to be *refractory* if TFI is < 60 day, while it is considered to be *sensitive* if TFI is ≥ 60 days.  *Refractory disease*  A response rate ≤ 5% would be considered of no further interest. According to the Fleming’s single stage design and assuming that the experimental treatment could guarantee a response rate ≥ 20%, for a 5% significance level and 80% power, 22 patients with *refractory* disease are needed to be enrolled into the study. If 4 or more objective responses will be observed it can be concluded that a response rate > 5% is possible.  *Sensitive disease*  A response rate ≤ 15% would be considered of no further interest. According to the Fleming’s single stage design and assuming that the experimental treatment could guarantee a response rate ≥ 30%, for a 5% significance level and 80% power, 43 patients with *sensitive* disease are needed to be enrolled into the study. If 11 or more objective responses will be observed it can be concluded that a response rate >15% is possible. |

# LIST OF ABBREVIATIONS

AE Adverse Event

ALT Alanine Aminotransferase

ANC Absolute Neutrophil count

AST Aspartate Aminotransferase

AUC Area Under the Curve

BSA Body Surface Area

CAV Cyclophosphamide/Doxorubicin/Vincristine

CR Complete Response

CRF Case Report Form

CRO Clinical Research Organization

CT Computerized Tomography

CTCAE Common Terminology Criteria for Adverse Event

DCR Disease Control Rate

DLT Dose Limiting Toxicity

ECG Electrocardiogram

ECOG Eastern Cooperative Oncology Group

EDC Electronic Data Capture

FDG-PET Fluorodeoxyglucose-Positron Emission Tomography

GCP Good Clinical Practice

IC Informed Consent

ICF Informed Consent Form

ICH International Conference of Harmonization

LCNEC Large Cell NeuroEndocrine Carcinoma

LD-50 Letal Dose 50

NSCLC Non Small Cell Lung Cancer

MRI Magnetic Resonance Imaging

MTD Maximum Tolerated Dose

ORR Objective Response Rate

OS Overall Survival

PD Progressive Disease

PFS Progression Free Survival

PI Principal Investigator

PLT Platelet

PR Partial Response

PS Performance status

RECIST Response Evaluation Criteria in Solid Tumors

SAE Serious Adverse Event

SCLC Small cell lung cancer

SD Stable Disease

SPARC Secreted Protein Acidic and Rich in Cysteine

TFI Treatment Free Interval

TTP Time To Progression

ULN Upper Limit of Normal

WBC White Blood Cell

WHO World Health Organization

# BACKGROUND AND INTRODUCTION

## Background

Despite high sensitivity to first-line chemotherapy, most SCLC patients eventually relapse.

Efficacy of second-line therapy is modest and highly influenced by the type and duration of response to prior chemotherapy.^1^ Topotecan is the only drug registered specifically for the treatment of relapsed SCLC owing it to the demonstration of superiority over placebo ^2^ and equivalence to CAV combination chemotherapy.^3^

However, topotecan modest and transient activity is counterbalanced by significant haematological toxicity and cumbersome schedule.^4^ Therefore, there is the need for more effective and better tolerated treatments.

Paclitaxel is among the other active agents for the treatment of SCLC, both alone and combined with carboplatin in first-line and second-line treatment. Particularly, combination of carboplatin and paclitaxel has been shown to be highly active even in *refractory* relapsed SCLC.^5–7^

Nab-paclitaxel is a solvent-free nanoparticle albumin-bound paclitaxel, which allows better tumor penetration and less toxicity.^8–11^ This drug has been found to be active in NSCLC and is registered for this indication in US.^10^ However, Nab-paclitaxel has not been formally studied in SCLC.

## 1.2 Nab-paclitaxel (Abraxane®)

### *1.2.1 Introduction*

Paclitaxel is a chemotherapy agent belonging to taxane family, which has been found to be active in various cancers. In Small Cell Lung Cancer (SCLC), it is active both as single agent and combined with carboplatin, in first- and second-line treatment. Moreover, paclitaxel combination with carboplatin has been shown to be highly active in *refractory* relapsed SCLC. ^5–7^

Since it is insoluble in water, paclitaxel needs a solvent, e.g. Cremophor EL®, which is cause of many solvent-related severe toxicities, such as anaphylactic hypersensitivity reactions and peripheral neuropathy.^12^ Nanoparticles Albumin-Bound (Nab)-paclitaxel (Abraxane®) is a new formulation of paclitaxel made through high-pressure homogenization of paclitaxel in presence of serum albumin. Through this process a 130 nm average diameter nanoparticle colloidal suspension at 3-4% concentration of paclitaxel is obtained. This formulation takes advantage of natural properties of albumin to reversibly bind paclitaxel, transport it across endothelial cells by glycoprotein 60 (gp-60)-mediated transcytosis and concentrate it inside tumor tissue, probably thanks to Secreted Protein, Acidic and Rich in Cysteine (SPARC).

Advantages of this formulation are reductions in reconstitution volume, infusion time, risk of hypersensitivity reactions, incidence of neutropenia, time needed to recover from peripheral neuropathy below grade 2 according to Common Terminology Criteria for Adverse Events (CTCAE), and increase in paclitaxel dose delivered to tumor tissue in comparison to solvent-based paclitaxel.^13^

Nap-paclitaxel is approved as single agent for the treatment of metastatic breast cancer; furthermore, it is approved as first-line therapy for metastatic pancreatic adenocarcinoma in combination with gemcitabine and for advanced NSCLC in combination with carboplatin (in US).

### *1.2.2 Preclinical experience*

Paclitaxel promotes tubulin polymerization and stabilizes microtubules in abnormal structures interfering in mitosis, cell motility and intracellular transport, and resulting in cytotoxic activity. Studies have shown in vivo activity in a multitude of different types of cancer.^14^

Nab-paclitaxel has been studied in nude mice bearing tumor xenografts from lung (H522), breast (MX-1), ovarian (SK-OV-3), prostate (PC-3), and colon (HT29) in comparison with solvent-based paclitaxel (sb-paclitaxel). Nab-paclitaxel has been found to be active and to cause tumor regression and prolonged survival in all of these models; sensitivity was maximal in lung, followed by breast, ovary, prostate and colon. The LD50 and maximum tolerated dose (MTD) in mice were 47 and 30 mg/kg/d (for a once a day infusion for 5 days) respectively. At doses bearing the same toxicity, groups treated with Nab-paclitaxel showed more complete regressions, longer doubling time and time to recurrence over than sb-paclitaxel ones, leading to a significant prolonged survival. At equal dose, intra-tumoral paclitaxel area under the curve (AUC) was 33% higher for nab-paclitaxel than sb-paclitaxel, indicating more effective intra-tumoral accumulation of the first one. Endothelial binding and transcytosis of paclitaxel were markedly higher for Nab-paclitaxel versus sb-paclitaxel.^15^

### *1.2.3 Phase I - II trials*

In a phase I study, nineteen patients, 13 of whom diagnosed with breast cancer and 6 with melanoma, were treated on an outpatients basis with a 30-minute infusion without premedication, with escalating dose levels. Dose Limiting Toxicities (DLTs) occurred at 375 mg/sqm q3w (6 pts treated at this dose level) and were sensory neuropathy, stomatitis and superficial keratitis (all of grade 3), so that MTD was set at 300 mg/sqm. Bone marrow suppression was mild with 7 out of 96 (7,3%) treatment cycles with nadir absolute neutrophil count (ANC) below 500/mmc, one of which with associated fever, and one case of platelet count dropping below 75.000/mmc (nadir 25.000/mmc), requiring the only platelet infusion needed in the study.^16^

Another phase I study explored the feasibility of Nab-paclitaxel weekly administration (d1, 8, 15 , 28-days cycles) in advanced solid tumors. Thirty-nine patients were enrolled and stratified in heavily pre-treated (HP) versus lightly pre-treated (LP) patients. Thirty-three % of patients received at least 6 cycles. DLTs occurred at 125 mg/sqm and 175 mg/sqm and consisted of neutropenia of grade 4 and sensory neuropathy of grade 3 in HP and LP, respectively. MTD was set at 100 mg/sqm and at 150 mg/sqm in HP and LP, respectively. No premedication was suggested and no hypersensitivity reactions occurred. Remarkably, solvent-free paclitaxel formulation has predictable pharmacokinetics, i.e. linear clearance, independently from dose, in the tested range of dose.^17^

Socinski et al. in 2010 explored optimal dose and schedule of first-line nab-paclitaxel in association with carboplatin in advanced NSCLC by a dose-finding phase I/II study. Nab-paclitaxel was administered at different doses and schedules (225mg/sqm q3w, 260mg/sqm q3w, 300mg/sqm q3w, 340mg/sqm q3w, at 140mg/sqm d1,8 q3w, 100mg/sqm weekly or 125mg/sqm weekly) in combination with carboplatin at AUC = 6 q3w in each of the 7 cohorts. The study population included 175 pts and safety and efficacy were the primary end points. All schedules showed some grade of activity; in particular, the cohort of pts treated at 140mg/sqm d1,8 q3w showed the highest disease control rate (DCR: 64%) and a higher grade 4 haematological toxicity than other cohorts (neutropenia: 44%; thrombocytopenia: 12%; anemia: 4%). Cohort of pts treated at 100mg/sqm weekly showed slightly lower DCR (56%) but a more favourable haematological toxicity profile (neutropenia of grade 4: 28%; thrombocytopenia of grade 4: 4%). Most common toxicities of grade ≥3 in all cohorts were neutropenia (60%), leukocytopenia (32%), thrombocytopenia (29%), anemia (21%), peripheral neuropathy (18,9%), fatigue (9%), myalgia (4.6%) and arthralgia (2,3%).^9^

### *1.2.4 Pharmacokinetics*

Paclitaxel plasmatic AUC following Nab-paclitaxel infusion rises in a linear way from 2.653 to 16.736 ng*hr/ml for doses from 80 to 300 mg/sqm.

In a study by Gardner et al, pharmacokinetics characteristics of paclitaxel following intravenous administration of Nab-paclitaxel 260 mg/sqm in 30 minutes in comparison with sb-paclitaxel injection at 175 mg/sqm were compared. Nab-paclitaxel clearance and distribution volume were 43% and 53% higher than sb-paclitaxel, respectively. No differences were demonstrated in terminal half-life.^18^

In another study enrolling twelve patients who received repeated Nab-paclitaxel administration at 260 mg/sqm, AUC varied by 19% (range 3.21-27.70%) among administrations in the same patient, without evidence of drug accumulation.

Paclitaxel has a high binding rate to plasma proteins, as assessed by mean of ultrafiltration. Free paclitaxel fraction following Nab-paclitaxel administration was significantly higher (6.2%) than after sb-paclitaxel (2.3%) resulting in a significantly higher drug exposure for Nab-paclitaxel, but a comparable total exposure possibly due to a higher clearance and distribution volume (1.741 L) of this formulation.^19^

According to published data on *in vitro* studies with human serum proteins, concomitant presence of cimetidine, ranitidine, dexamethasone or diphenhydramine does not affect paclitaxel protein binding capacity.

Paclitaxel is mainly eliminated after liver transformation: its main metabolite is 6a-hydroxypaclitaxel; 3’-p-hydroxypaclitaxel and 6a-3’-p-hydroxypaclitaxel are quantitatively minor metabolites. Metabolites result from the action of microsomal enzymes CYP2C8, CYP3A4 and CYP2C8 together with CYP3A4 respectively.^20^

At doses from 80 mg/sqm to 300 mg/sqm, commonly used in clinical practice, plasmatic clearance range goes from 13 to 30 L/h*sqm and terminal half-life from 13 to 27 hours.

How liver function impairment affects Nab-paclitaxel has been studied in patients with advanced solid tumors. Patients with normal liver function (n=130), mild (n=8), moderate (n=7) and severe (n=5) impairment according to NCI Organ Dysfunction Working Group^21^ were included into the study. Analyses showed that mild liver dysfunction (total bilirubin 1-1.5 x ULN) have no clinically relevant effects, while patients with moderate (total bilirubin 1.5-3 x ULN) and severe (total bilirubin 3-5 x ULN) liver dysfunction have a 22-26% decrease in paclitaxel elimination maximum speed resulting in a 20% increase in the AUC, in comparison with patients with normal liver function.

No correlation has been found between liver dysfunction and neutropenia if Nab-paclitaxel dose was adjusted accordingly.

### *1.2.5 Phase III trials*

First Nab-paclitaxel approval came in 2005 following the results from a phase III clinical trial including 454 metastatic breast cancer (mBC) patients. Patients were randomly assigned to receive either Nab-paclitaxel 260 mg/sqm every 3 weeks without premedication (n = 229) or sb-paclitaxel 175 mg/m2 every 3 weeks with premedication (n = 225). Nab-paclitaxel demonstrated significantly higher response rates (33% vs. 19%; p = .001) and longer time to tumor progression (23.0 vs. 16.9 weeks; HR 0.75; p= .006) compared with sb-paclitaxel. The incidence of grade 4 neutropenia was significantly lower for Nab-paclitaxel than sb-paclitaxel (9% vs. 22%, respectively; p < .001) despite a 49% higher paclitaxel dose. Febrile neutropenia was uncommon (< 2%), and the incidence did not differ between the two treatment arms. Grade 3 sensory neuropathy was more common in the Nab-paclitaxel arm than in the standard paclitaxel arm (10% vs. 2%, respectively) but was easily managed and improved rapidly (median, 22 days). No hypersensitivity reactions occurred with nab-paclitaxel despite the absence of premedication and shorter administration time.^22^

Nab-paclitaxel approval as first-line treatment for metastatic pancreatic adenocarcinoma was due to results from an international multicentre, open-label randomized phase III trial. Gemcitabine 1000 mg/sqm was administered on a weekly basis with or without Nab-paclitaxel 125 mg/sqm d1, 8, 15, 29, 36, 43 on first cycle and then d1, 8, 15 q28 for subsequent cycles. Eight hundred and sixty-one patient were enrolled, 431 in the experimental arm and 430 in the control. Nab-paclitaxel arm showed a longer median OS (mOS), the primary endpoint, in comparison with gemcitabine alone (8.5 vs. 6.7 months, HR 0.72, p < .001) and a longer median PFS (mPFS, 5.5 vs. 3.7 months, HR 0.69, p < .001). In the experimental arm there were also a significantly higher overall response rate (23% vs. 7%; p < .001) and disease control rate (48% vs. 33%; p < .001) according to independent review. In Nab-paclitaxel arm, the most common treatment-related grade 3 or higher hematological adverse events were neutropenia (36%) and leukopenia (31%); febrile neutropenia incidence was 4%. Grade 3 peripheral neuropathy occurred in 17% of patients (no grade 4 peripheral neuropathy occurred) and the median time to improvement to grade ≤1 was 29 days.^23^

Clinical utility of Nab-paclitaxel in lung cancer was first explored in NSCLC in 2012. Socinski et al. conducted a multicentre, randomized, phase III study enrolling 1052 patients with advanced, untreated NSCLC to receive carboplatin AUC = 6 q3w in combination with either Nab-paclitaxel 100 mg/sqm d1, 8, 15 q21 (n=514) or sb-paclitaxel 200 mg/sqm q3w (n=524). Primary endpoint was ORR and the study was powered to recognize a 40% ORR improvement in Nab-paclitaxel-treated arm with 80% statistical power. Overall response rate was higher in Nab-paclitaxel arm versus sb-paclitaxel one (33% vs. 25%; RR ratio 1.313, p = .005). According to histology, squamous-NSCLC (Sq-NSCLC) patients treated with nab-paclitaxel had a higher ORR than those treated with sb-paclitaxel (41 vs. 24%; RR ratio 1.680, p < .001); conversely no significant difference was seen in non-squamous NSCLC (NSq-NSCLC) patients (ORR 26 vs. 25%; p = .808). In a subsequent data analysis based on the pre-planned strata, no significant difference was demonstrated in terms of OS and PFS among histology subgroups, albeit the confirmed ORR benefit in Sq-NSCLC vs. NSq-NSCLC.^11^ There was no significant improvement both in mOS (12.1 vs. 11.2 months, HR 0.922; p= 0.27) and in mPFS (6.3 vs. 5.8 months, HR 0.902; p =0.214) in Nab-paclitaxel vs. sb-paclitaxel groups, respectively. Furthermore, Nab-paclitaxel showed OS benefit in comparison with sb-paclitaxel among North Americans and pts older than 70 years.

Nab-paclitaxel showed a more favourable toxicity profile in terms of peripheral neuropathy (3 vs. 12%), neutropenia (47 vs. 58%), arthralgia (0 vs. 2%) and myalgia (<1 vs. 2%), all of grade ≥3. On the other hand, there was an increased incidence of anaemia (27 vs. 7%) and thrombocytopenia (18 vs. 9%) in Nab-paclitaxel than in sb-paclitaxel group.

Any grade sensory neuropathy grade occurred significantly more often in the Nab-paclitaxel arm (46 vs. 62%). Median time needed to grade ≥3 sensory neuropathy to improve to grade ≤1 was 38 days in the Nab-paclitaxel arm vs. 104 days in the sb-paclitaxel arm.^10^

Safety and efficacy of Nab-paclitaxel in SCLC have not been extensively studied. In a non-comparative phase II trial, 27 chemo-naive ED-SCLC pts were randomized in a 1:1 ratio to receive carboplatin AUC = 6 either in combination with Nab-paclitaxel 300 mg/sqm q3w (arm A; 14 pts) or Nab-paclitaxel 100 mg/sqm d1, 8, 15 q21 (arm B; 13 pts). The study had also been amended to reduce Nab-paclitaxel dose to 240 mg/sqm in arm A and 80 mg/sqm in arm B, due to significant number of dose reductions in both arms and/or omissions of day 8 or 15 in arm B. The study was stopped early because of a slow accrual; hence, the impact of dose reduction on toxicity and compliance could not be assessed.

The most common treatment related grade ≥ 3 toxicities in both arms were neutropenia (58% arm A, 38% arm B), anaemia (29% arm A, 31% arm B) and thrombocytopenia (21% arm A, 23% arm B). No sensory neuropathy of grade≥ 3 in the weekly Nab-paclitaxel arm was seen. No conclusion could be done on ORR, primary endpoint, due to small sample size.^24^

### *1.2.6 Safety profile*

Most common reported drug related adverse events with Nab-paclitaxel single agent are neutropenia, peripheral neuropathy, arthralgia, myalgia, gastrointestinal symptoms and fatigue, as reported in the most updated version of investigator brochure (IB).

**Neutropenia**: although it is less common than sb-paclitaxel, neutropenia of grade ≥ 3 with Nab-paclitaxel occurred variably from 9 to 60% in different phase I-III trials, resulting in some of them as DLT. It was reversible and dose-related. Incidence of febrile neutropenia was around 1-4%. Overall incidence was higher when Nab-paclitaxel was associated with other chemotherapy agents, e.g. gemcitabine or carboplatin. There is no evidence of cumulative bone marrow toxicity.

Subjects with febrile neutropenia or infection should be evaluated promptly and treated with appropriate antibiotic therapy and/or therapeutic granulocyte colony stimulating factor as local standards of care.

**Other hematological AEs**: Anaemia and thrombocytopenia are more frequent with Nab-paclitaxel than sb-paclitaxel, and grade 3 or higher occurred in up to 18 and 28% of patients treated with clinical adopted schedules, respectively. Packed red blood cells and platelets transfusions should be administered as clinically indicated. Erythropoietic agents such as epoetin alfa or darbepoetin alfa may be used at the physician discretion. Grade 3 or higher leukopenia is reported in up to 50% of cases. Bone marrow toxicity is reversible and there is no evidence of cumulative toxicity effect.

**Peripheral Neuropathy**: peripheral neuropathy is reported in up to 68% of pts treated with Nab-paclitaxel, mainly as sensory neuropathy, hypoesthesia or paraesthesia, but motor neuropathy is not rare. Reported median time to neuropathy onset is 140 days and it was generally of grade 1 or 2. Incidence and severity are dose and schedule dependent. Neuropathy is mostly reversible at Nab-paclitaxel suspension and median time to improvement to grade 1 or lower is between 22 and 29 days but can be as long as 66 days, depending on schedule; once symptoms have improved, treatment could be resumed. No grade 4 neuropathies have been reported.

**Arthralgia/myalgia**: Any grade arthralgia and/or myalgia occurred in about 30% and 24% of pts, respectively, grade 3 or higher in about 6-7% of pts, less common in weekly administration schedules, such as the one used in NSCLC (incidence below 1%). It usually starts 3 days after Nab-paclitaxel administration and improves in 7 days on median. Symptoms can be managed with non-steroidal anti-inflammatory agents and acetominophene.

**Gastrointestinal Signs/Symptoms**: Main gastrointestinal AEs reported are diarrhea and nausea, in 25-30% of pts, followed by vomiting (20-25%), stomatitis (15-20%) and constipation (5%). Grade 3 or higher AEs are uncommon. Supportive care for these events may include premedication with antiemetic drugs to limit potential recurrent nausea and vomiting, loperamide or laxatives as needed. Due to stomatitis occurrence, supportive oral care products and analgesics may be warranted.

**Fatigue**: Fatigue of any grade is reported in up to 40% of pts, grade 3 or higher AEs occur in about 3% of pts, but no dose reduction or delay is reported in literature.

**Alopecia**: hair loss has been reported in over 80% of pts and high-grade alopecia is expected in more than 50% of pts. Hair loss generally occurred within a month after treatment start.

**Hypersensitivity reactions**: Nab-paclitaxel could be safely administered as a 30-minute infusion without dexamethasone or antihistaminic premedication. No severe hypersensitivity reactions were reported. In case of hypersensitivity reactions, infusion should be stopped immediately, symptomatic therapy should be administered and Nab-paclitaxel discontinued definitively.

**Eye toxicity**: In earlier trials, different sight alterations were reported (blurred or smoky vision, light flashes, photosensitivity) and ocular keratopathy up to grade 3, which was DLT in a cohort of pts. In case of keratopathy, a full ophthalmologic evaluation and topical lubricating drop are recommended. No patient developed permanent ocular sequelae.

**Pneumonitis**: Pneumonitis occurred in about 1% and 4% of pts treated with Nab-paclitaxel as monotherapy and in association with gemcitabine, respectively. Patients with history of slowly progressive dyspnoea and unproductive cough, or pulmonary conditions such as sarcoidosis, silicosis, idiopathic pulmonary fibrosis, hypersensitivity pneumonitis or multiple allergies should not be enrolled. Patients should be carefully evaluated for the presence of signs or symptoms of pneumonitis or transient and repeated dyspnoea with unproductive persistent cough and fever. Chest X-rays or CT scans may be indicated to look for infiltrates, ground-glass opacities, or honey combing patterns. Once infective aetiology has been ruled out by routine microbiological and/or immunologic methods, Nab-paclitaxel should be suspended definitively and supportive intravenous high dose corticosteroid therapy promptly started, with secondary pathogen coverage. Appropriate oxygen or ventilation support should be used when required.

**Sepsis**: Episodes of sepsis, in up to 5% of pts, have been reported mainly in pts affected by pancreatic adenocarcinoma and receiving Nab-paclitaxel together with gemcitabine, independently of ANC. In case of fever, large spectrum antibiotic therapy should be administered, irrespective of ANC.

## 1.3 Rationale of the study

For patients with relapsed SCLC after a platinum-based first-line chemotherapy, treatment options and clinical outcomes are very poor. Topotecan or CAV regimens are the available therapeutic options in this patient population.

These therapeutic options have limited efficacy and are burdened by significant toxicity. Therefore, there is an urgent need to explore other drugs characterized by acceptable clinical activity and toxicity profiles. Paclitaxel, both as single agent and combined with carboplatin, has shown a significant clinical activity in chemo-naïve, relapsed and also in *refractory* SCLC patients. However, its use in clinical practice is limited due to a high risk of anaphylactic reactions and peripheral neurotoxicity.

Nab-paclitaxel, a solvent-free nanoparticle albumin-bound paclitaxel, has been registered for the treatment of several solid tumors, including also untreated advanced NSCLC for which it has demonstrated a higher tumor penetration and a better toxicity profile over paclitaxel.

Taking into account that Nab-paclitaxel has not been formally studied in SCLC, this study is intended to explore clinical activity and safety profile of this compound in patients with *sensitive* or *refractory* SCLC relapsed after first-line platinum-based chemotherapy. The schedule and dose chosen in the proposed study have shown a favourable risk/benefit profile and activity, as in other malignancies, including also NSCLC.

# TRIAL DESIGN

This is an open label, multicentre, phase II study evaluating the activity and safety of Nab-paclitaxel in patients with *sensitive* or *refractory* SCLC and large-cell neuroendocrine carcinoma (LCNEC) who have relapsed after first-line platinum-based chemotherapy. It is planned to recruit approximately 65 subjects at up to 27 national centers.

Two patients’ cohorts will be enrolled: *refractory* (22 patients) and *sensitive* (43 patients) respectively; a Fleming’s one stage design will be applied to each cohort to decide if Nab-paclitaxel response rate (the percentage of patients with confirmed complete response or partial response) is sufficient to justify further investigation of the drug in SCLC patients.

Patients will be classified according to treatment free interval (TFI), i.e. the interval from the last chemotherapy administration during first-line therapy and the occurrence of progressive disease. Disease is considered to be *refractory* if TFI is < 60 day, while it is considered to be *sensitive* if TFI is ≥ 60 days.

Eligible patients will be registered to receive Nab-paclitaxel (30-min infusion) 100 mg/sqm weekly on days 1, 8, 15 q 28 days. Chemotherapy will be continued until a maximum of 6 courses or progressive disease or intolerable toxicity or patient refusal. Disease evaluation will be assessed every 2 cycles (every 8 weeks ± 1) until documented progression or end of treatment. In patients with confirmed and prolonged disease response, clinical benefit and good tolerance to study drug treatment, the investigators can evaluate to continue therapy beyond 6^th^ cycle, after discussion with Principal Investigator (PI) of the study. Patients who end treatment without evidence of progressive disease, will be evaluated for disease status every 8 weeks.


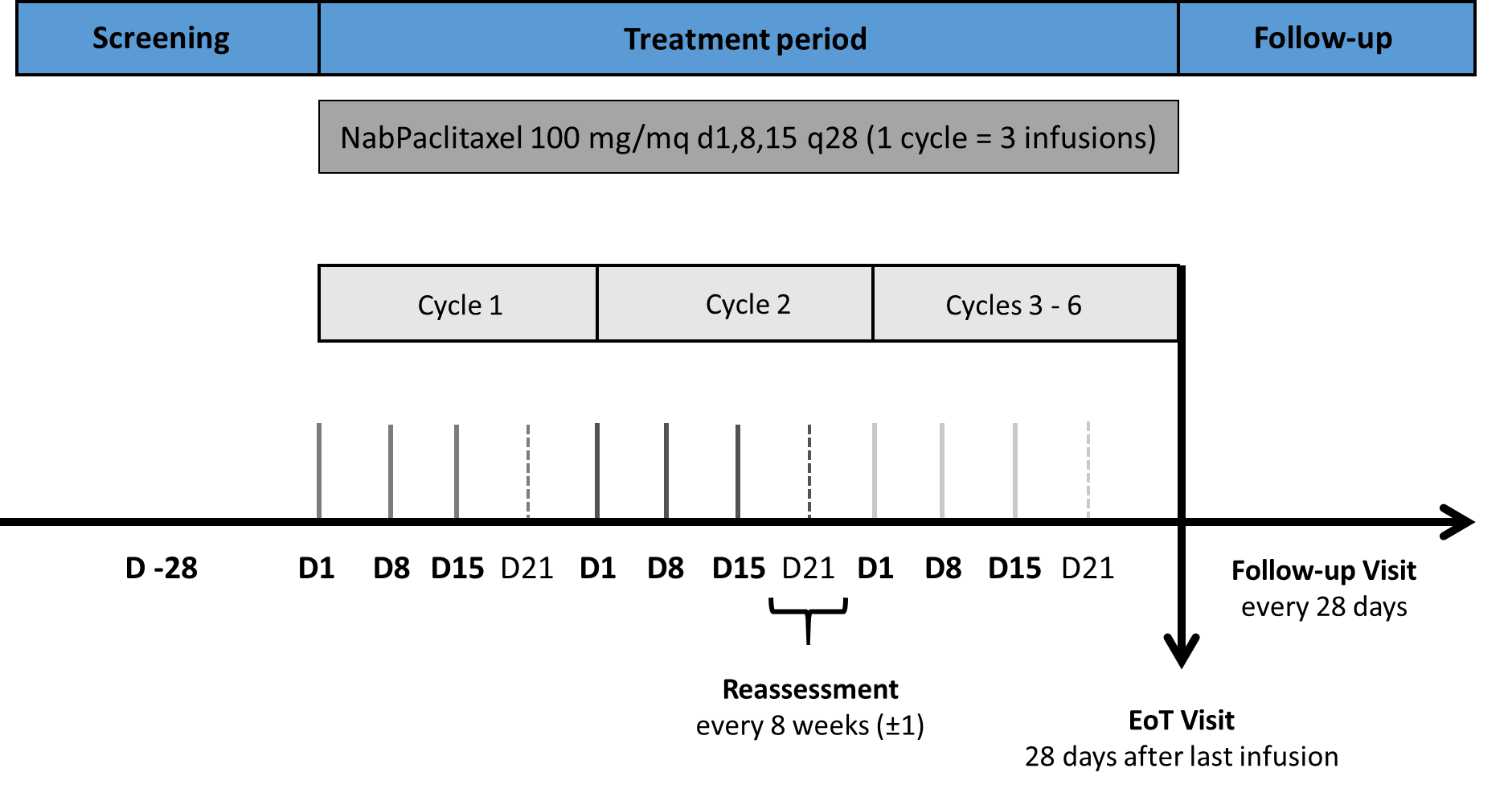


# OBJECTIVES AND END-POINTS

##

## 3.1 Primary objective

To evaluate the therapeutic activity of Nab-Paclitaxel in subjects with *sensitive* and *refractory* small cell lung cancer (SCLC) and large-cell neuroendocrine carcinoma (LCNEC) relapsed after cisplatin or carboplatin and etoposide first line chemotherapy.

## 3.2 Secondary objectives

To evaluate safety and efficacy of Nab-Paclitaxel in subjects with *sensitive* and *refractory* small cell lung cancer (SCLC) and large-cell neuroendocrine carcinoma (LCNEC) relapsed after cisplatin or carboplatin and etoposide first line chemotherapy.

## 3.3 Primary End-point

The primary end-point is objective tumor response. Tumor responses will be evaluated according to standard RECIST 1.1 criteria and will be based on the Investigator’s assessment. Data will be reported as percentage of complete responses (CRs), partial responses (PRs), stable disease (SD) and progressive disease (PD). Exact binomial method will be used to estimate the response rate (CR+PR) and its 95% confidence interval. Patients with no tumor assessment after baseline will be classified as non-responders.

## 3.4 Secondary End-points

The secondary end-points are:

1. Toxicity: the assessment of safety will be based mainly on the frequency of adverse events; toxicity will be measured according to NCI Common Toxicity Criteria Adverse Event (CTCAE), version 4.03.
2. Progression Free Survival (PFS) will be calculated from the patient registration to the evidence of progressive disease, or death, or the last date the patient was known to be progression-free or alive.
3. Overall Survival (OS) will be calculated from the registration to death from any cause, or the last date the patient was known to be alive.

# STUDY POPULATION

## 4.1 Inclusion criteria

Study subjects must meet all of the following criteria to be considered for inclusion:

- Pathologically (histology or cytology) confirmed diagnosis of small cell lung cancer (SCLC) or large-cell neuroendocrine carcinoma (LCNEC) or poorly differentiated (G3) neuroendocrine cancer of the lung (according WHO classification 2015)
- Male or female and ≥ 18 years of age
- Life expectancy ≥ 12 weeks
- Have progressed after or during platinum-based standard chemotherapy regimen (cisplatin or carboplatin and etoposide) for first-line treatment of SCLC, either limited stage (LD) or extensive stage (ED) disease and have not received any other treatment (except for immunotherapy as maintenance treatment), including re-treatment with front-line regimen
- Have measurable disease per Response Evaluation Criteria in Solid Tumors, version 1.1 (RECIST 1.1); clear radiological evidence of disease progression after first-line therapy has to be documented; No previous RT on the only site of measurable or evaluable disease, unless that site had subsequent evidence of progression
- Eastern Cooperative Oncology Group (ECOG) performance status (PS) of 0 or 1
- Patients with treated brain metastases with stable lesions for at least 2 weeks and off steroids or on a stable dose of steroids. Radiotherapy must have been completed a minimum of 14 days prior to registration, and patient must have recovered from AEs related to radiotherapy to < grade 1 (except alopecia).
- For Females: must be postmenopausal (defined as occurring 12 months after last menstrual period) before the screening visit, or are surgically sterile. If they are of childbearing potential, a negative serum pregnancy test prior to study entry has to be documented; furthermore, they agree to practice 2 effective methods of contraception, at the same time, from the time of signing the informed consent form (ICF) through 30 days after the last dose of study drug, or agree to practice true abstinence, when this is in line with the preferred and usual lifestyle of the subject
- For Males: even if surgically sterilized (i.e. post-vasectomy status) agree to practice effective barrier contraception during the entire study treatment period and through 6 months after the last dose of study drug, or practice true abstinence, when this is in line with the preferred and usual lifestyle of the subject.
- Screening clinical laboratory values as specified below:
  - Absolute neutrophil count (ANC) ≥ 1500/mm3, platelet count ≥ 100,000/mm3, and haemoglobin ≥ 9 g/dL
  - Total bilirubin < 1.5 the institutional upper limit of normal (ULN)
  - Serum alanine aminotransferase (ALT) or aspartate aminotransferase (AST) < 2.5 the institutional ULN (< 5 if liver function test elevations are due to liver metastases)
  - Creatinine < 1.5 institutional ULN or estimated creatinine clearance using the Cockcroft-Gault formula ≥ 30 mL/minute for patients with creatinine levels above institutional limits
- Stable medical condition, including the absence of acute exacerbations of chronic illnesses, serious infections, or major surgery within 4 weeks before registration, and otherwise noted in other inclusion/exclusion criteria
- Recovered (i.e., ≤ Grade 1 toxicity) from effects of prior anticancer therapy, except alopecia
- Prior radiotherapy is allowed provided that it has been completed more than 2 weeks before starting Nab-paclitaxel
- Ability to comply with protocol requirements
- The patient or the patient’s legal representative has to be able to provide written informed consent. Voluntary written consent must be given before performance of any study-related procedure not part of standard medical care, with the understanding that the patient may withdraw consent at any time without prejudice to future medical care.

## 4.2 Exclusion criteria

Patients meeting any of the following exclusion criteria are not eligible to the study:

- Any prior not platinum-based chemotherapy treatment for SCLC or large-cell neuroendocrine carcinoma (LCNEC ) (immunotherapy is allowed as maintenance treatment)
- Prior treatment with Nab-paclitaxel, paclitaxel or any other taxane agent
- Known hypersensitivity to Cremophor® EL, paclitaxel, or its components
- Any comorbid condition or unresolved toxicity that would preclude administration of weekly Nab-paclitaxel
- Prior history of Grade ≥ 2 neurotoxicity that is not resolved to ≤ Grade 1
- Patients with symptomatic and/or progressive brain metastases or with carcinomatous meningitis
- Diagnosed with or treated for another malignancy within 3 years before the first dose of study drug, or previously diagnosed with another malignancy and have any evidence of residual disease. Patients with non-melanoma skin cancer or carcinoma in situ of any type may be enrolled in the study if they have undergone complete resection and no evidence of active disease is present
- History of myocardial infarction, unstable symptomatic ischemic heart disease, uncontrolled hypertension despite appropriate medical therapy, any ongoing cardiac arrhythmias of Grade > 2, thromboembolic events (e.g., deep vein thrombosis, pulmonary embolism, or symptomatic cerebrovascular events), or any other cardiac condition (e.g., pericardial effusion or restrictive cardiomyopathy) within 6 months before receiving the first dose of study drug. Chronic stable atrial fibrillation on stable anticoagulant therapy is allowed. Patients with a pacemaker may be enrolled in the study upon discussion with the project clinician
- Infection requiring IV antibiotic therapy or other serious infection within 14 days before the first dose of study drug
- For female subjects: positive serum pregnancy test, pregnancy or breast feeding
- Surgery within 3 weeks (or 2 weeks for a minor surgery) before study enrolment and not fully recovered to baseline or to a stable clinical status. Insertion of a vascular device is allowed
- Unwilling or unable to comply with the protocol or cooperate fully with the investigator and site personnel.

# 5. DRUG AND SCHEDULE, TOXICITY AND DOSE MODIFICATIONS

## 5.1 Nab-paclitaxel (Abraxane®): drug, initial dose and schedule

Nab-paclitaxel will be administered through a 30-min infusion at 100 mg/sqm weekly on days 1, 8, 15 q28 days.

Study drug will be packaged by Celgene according to all local legal requirements. Study drug will be labelled in accordance with applicable regulatory requirements. All study drug supplies must be stored in accordance with Celgene instructions. Storage conditions for the study drug will be described on study drug label. Until dispensed to the subjects, the study drug will be stored in a securely locked area, accessible to authorized personnel only. The Investigator is responsible for maintaining accurate drug accountability records throughout the study. Each IV infusion of study drug will be documented in the electronic data capture (EDC) system.

The Investigator is responsible for destroying all unused study drug to the site. The Sponsor or designee must verify that no remaining supplies are in the Investigator’s possession at the end of the study, unless otherwise instructed by the Sponsor.

**Supplier**

Celgene will supply Nab-paclitaxel for the entire duration of the study. Celgene will send Nab-paclitaxel to the central Sponsor pharmacy depot. The Sponsor pharmacy depot will dispense the drug to participating centers.

**Dosage form**

Abraxane - powder for suspension for infusion - will be supplied in vials and each vial contains 100 mg of paclitaxel (as paclitaxel albumin).

After reconstitution, each ml of suspension contains 5 mg of paclitaxel (as paclitaxel albumin). Excipients when reconstituted, each ml of Abraxane concentrate contains 0.183 mmol sodium, which is 425 mg of sodium per dose.

**Storage and Handling**

Nab-paclitaxel is a cytotoxic anticancer drug and caution should be exercised in handling Nab-paclitaxel. The use of gloves is recommended.

If Nab-paclitaxel (lyophilized cake or reconstituted suspension) comes in contact with the skin, the skin should be washed immediately and thoroughly with soap and water.

Dyspnea, chest pain, burning eyes, sore throat and nausea have been reported after inhalation of solvent-based paclitaxel. If Nab-paclitaxel comes in contact with mucous membranes, the membranes should be flushed thoroughly with the water.

**Stability**

*Stability of unopened vials.* Unopened vials of Nab-paclitaxel are stable until the date indicated on the package when stored in the original cartoons at USP controlled room Temperature (25, however excursion are permitted within 15 -30). Neither freezing nor refrigeration adversely affects the stability of Nab-paclitaxel.

*Stability of reconstituted suspension the vials.* The reconstituted suspension in the vials should be filled into an infusion bag immediately, to prevent risk of microbial contamination. If not filled immediately, storage time and conditions should not normally be longer than 8 hours at 2-8. If not use immediately, each vials of reconstituted suspension should be replaced in the original cartons to protect it from bright light.

*Stability of infusion suspension in the infusion bag.* The reconstituted suspension in the infusion bag should be used immediately to prevent risk of microbial contamination. If not used immediately storage time should not be longer than 8 hours at ambient temperature (approximately 25) and lighting conditions.

**Packaging**

Celgene will ship the investigational product to the Sponsor pharmacy depot in vials packaged in boxes. Each vial is labelled according to the information given by the Sponsor.

**Receipt of the drug**

The Investigator or designee is responsible for taking an inventory of each shipment of study drug received and comparing it with the accompanying study drug accountability form. The Investigator will verify the accuracy of the information on the form, sign and date it, retain a copy in the study file, and return a copy to Sponsor or its representative. Product Supplies shall be ordered by the Sponsor through the Celgene Drug Ordering System.

**Unused study drug supplies**

Unused study drug will be destroyed locally in compliance with local pharmacy destruction procedures and drug disposition must be appropriately documented in the study file. The destruction documents will be shipped to Celgene. If any study drug is lost or damaged, its disposition should be documented in the source documents.

## 5.2 Treatment duration

Treatment should be administered until a maximum of 6 courses, documented disease progression, unacceptable toxicity, or patient refusal.

In case of clear-cut progression occurring before the first disease evaluation, the treatment will be discontinued and the response to treatment will be assessed as "early progression".

In case of stable disease, the patient will continue the treatment until disease progression or a maximum of 6 courses.

In case of objective (complete or partial) response, the treatment will be continued until documented disease progression or maximum planned cycle numbers. The disease status will be regularly assessed every 2 courses (every 8 weeks ± 1 week) during this period to evaluate the duration of response.

In case of patient with confirmed and prolonged response, clinical benefit and good tolerance after 6 cycles, the investigators can consider the possibility to continue the experimental treatment beyond 6^th^ cycle, until progressive disease or cumulative toxicity or request of the patient to withdraw after discussion with Principal Investigators of the study.

## 5.3 Withdrawal criteria

Whatever the disease status, the treatment will be always discontinued in case of

- patient refusal;
- Excessive toxicity precluding further therapy, according to the responsible physician.

Patients discontinuing therapy in the absence of progression should not receive any other cancer treatment before their disease progresses, unless this is clearly not in the interest of the patient. After progression, the treatment will be left to the discretion of the treating physician. Any anti-cancer therapy other than the study drug given as single agent will not be considered as part of the protocol treatment.

## 5.4 Dose and schedule modifications

Patients will be monitored for toxicity at each drug administration and Nab-paclitaxel dose will be adjusted accordingly. Toxicity will be evaluated according to National Cancer Institute (NCI) Common Terminology Criteria for Adverse Events (CTCAE, version 4.03).

### *5.4.1 Day 1 of each cycle (from second cycle and beyond)*

*Haematological Toxicity*

For neutropenia (ANC < 1.500/mm3) or thrombocytopenia (platelet count < 100.000/mm3) on Day 1 of each cycle, treatment is to be delayed until recovery of ANC and platelet count above these values.

If recovery of haematological toxicity does not occur within 3 weeks, treatment has to be permanently discontinued

In presence of febrile neutropenia or delay of next cycle due to persistent neutropenia, Nab-paclitaxel dose has to be reduced by one dose level (75 mg/sqm) in case of first occurrence or second dose level if it is second occurrence (50 mg/sqm) or discontinue treatment in case of third occurrence. For thrombocytopenia < 50.000/mm3 at any time during prior cycle, Nab-paclitaxel dose has to be reduced by one dose level (75 mg/sqm) in case of first occurrence or second dose level if it is second occurrence (50 mg/sqm) or discontinue treatment in case of third occurrence (Table 1).

In case of febrile neutropenia, granulocyte colony stimulating factors (G-CSF) can be given based on standard of care local guidelines. G-CSF should not be given as neutropenia prophylaxis

Patients who experience grade 3-4 toxicity of any type despite 2 consecutive dose reductions of nab-paclitaxel should discontinue protocol treatment.

*Non-Haematological Toxicity*

All non-haematological toxicities have to be recovered to < grade 1, to allow treatment delivery on day 1 of each cycle. If toxicity recovery does not occur within 3 weeks, treatment has to be permanently discontinued.

Patients who experience grade 3-4 toxicity of any type despite 2 consecutive dose reductions of nab-paclitaxel should discontinue protocol treatment.

*Gastrointestinal Toxicity*

For nausea/vomiting of grade ≥ 3 despite anti-emetic therapy or stomatitis/mucositis grade ≥ 3 or diarrhoea grade ≥ 3, at any time during prior cycle, the dose of Nab-paclitaxel has to be reduced by 1 dose level (75 mg/sqm) (Table 1).

*Neurologic Toxicity*

For neuropathy of grade ≥ 3, Nab-paclitaxel dose has to be held until neuropathy is grade 1 or lower, and the dose has to be reduced by 1 dose level, when treatment is resumed (Table 1).

### *5.4.2 Day 8, 15 of each cycle*

If ANC >1.5 x 10^9/L AND PLT > 100 x 10^9/L AND non-haematological toxicity grade < 1. No dose change.

If ANC 1.0 - 1.5 x 10^9/L AND/OR PLT 75 - 100 x 109/L (days 8, 15): reduce Nab-paclitaxel to Dose Level -1 (75 mg/sqm)*.

If ANC < 1.0 x 10^9/L AND/OR PLT < 75 x 10^9/L (day 8): omit the dose, then reduce nab-paclitaxel to Dose Level –1 (75 mg/sqm) the next dose on day 15*.

If ANC < 1.0 x 10^9/L AND/OR PLT < 75 x 10^9/L (day 15): omit the dose then follow the specific guidelines for dose reductions at day 1 of the next cycle.

If non-haematological toxicity grade 2 on day 8: reduce nab-paclitaxel to Dose Level -1 (75 mg/sqm)*, on day 8 and 15*.

If non-haematological toxicity grade 2 on day 15: reduce nab-paclitaxel to Dose Level -1 (75 mg/sqm)*, then follow the specific guidelines for dose reductions at day 1 of the next cycle.

If non-haematological toxicity grade 3 or higher on day 8: omit the dose, then reduce nab-paclitaxel to Dose Level -1 (75 mg/sqm)* the next dose on day 15.

If non-haematological toxicity grade 3 or higher on day 15: omit the dose, then follow the specific guidelines for dose reductions at day 1 of the next cycle.

*Or Dose Level -2 (50 mg/sqm), if second occurrence or discontinue treatment if is third occurrence. Dose reductions adopted have to be maintain at all following nab-paclitaxel administrations.

The following table summarizes recommended dose modification for nab-paclitaxel treatment according to its associated toxicity:

| **Nab-paclitaxel** | | | |
| --- | --- | --- | --- |
| ***Dose Level*** | ***Dose*** | ***Dose reduction to mg/sqm*** | ***Amount Reduced*** |
| 1 | 100 mg/sqm | 75 mg/sqm | 25 mg/sqm |
| -1 | 75 mg/sqm | 50 mg/sqm | 25 mg/sqm |
| -2 | 50 mg/sqm | NA | NA |

**Table 1.** Permanent dose reductions on day 1 of each cycle (from second cycle and beyond) for hematologic and non-hematologic toxicities occurred during the prior cycle

| **Dose modification During Treatment** | | |
| --- | --- | --- |
| **Adverse Drug Reaction** | **Occurrence** | **Nab-paclitaxel Dose (mg/m²)** |
| Febrile neutropenia (ANC < 500/mmc with fever > 38°C)  OR  Delay of next cycle by >7 days for ANC < 1500/mmc  OR  ANC < 500/mm3 for > 7 days | First | 75 |
|  | Second | 50 |
|  | Third | Discontinue Treatment* |
| Platelets count < 50.000/mm3 | First | 75 |
|  | Second | 50 |
|  | Third | Discontinue Treatment* |
| Peripheral Neuropathy Grade ≥ 3 | First | 75 |
|  | Second | 50 |
|  | Third | Discontinue Treatment* |
| Grade 3-4Nausea/Vomiting  Grade 3-4 Diarrhea  Grade 3-4 Mucositis  Any other Grade 3 or 4 non-hematologic toxicity or other investigator defined unacceptable toxicity | First | 75 |
|  | Second | 50 |
|  | Third | Discontinue Treatment* |
|  |  |  |
|  |  |  |
| *ANC = Absolute Neutrophil Count; NA = Not Applicable*  **If an adverse event requiring dose reduction recurs after the dose has been reduced twice, the subject should generally have treatment discontinued unless, at the discretion of the investigator, there is evidence of continuing benefit to the subject that outweighs the risk of recurrence toxicity* | | |

# 6. CONCOMITANT TREATMENTS

Every medication or treatment taken by the patient during the trial and the reason for its administration must be recorded on the CRF.

## 6.1 Drugs with proarrhythmic potential

Concomitant treatment with a drug having proarrhythmic potential (terfenadine, quinidine, procainamide, disopyramide, sotalol, probucol, bepridil, haloperidol, risperidone, indapamide and flecainide) is not allowed during the study.

## 6.2 Inhibitors and inducers of CYP3A4 and CYP2C8

Paclitaxel is mainly eliminated after transformation by microsomal enzymes CYP3A4 and CYP2C8 (v. 1.2.4). Concomitant administration of known inhibitors (e.g. ketoconazole and itraconazole and other imidazole-derived antifungal agents, fluoxetine, gemfibrozil, cimetidine, ritonavir, saquinavir, indinavir e nelfinavir, clarithromycin, erythromycin, diltiazem, verapamil, delavirdine, atazanavir) or inducers (e.g. rifampicin, rifabutin, carbamazepine, phenobarbital, phenytoin, St. John’s wort, efavirenz, tipranavir, nevirapine) of CYP2C8 or CYP3A4 iso-enzymes is not recommended.

Alternative therapies should be used when available. If usage of a potent CYP3A4 inhibitor or inducer is necessary, this must be in agreement with the Sponsor.

## 6.3 Anticoagulants

The use of Coumarin-derivative anticoagulants such as warfarin (Coumadin®) should be avoided, if possible, because of the potential drug-drug interaction. In case of concomitant use of the two drugs, a more frequent monitoring of coagulation parameters is recommended.

## 6.4 Supportive care in case of toxicity or palliative radiotherapy

Patients should receive full supportive care during the study. Prophylactic use of hematopoietic growth factors to support neutrophil count or haemoglobin concentration is not recommended in the first 3 weeks of treatment and may be used subsequently at discretion of the treating physician and according to local guidelines.

Patients who enter the study on stable doses of erythropoietin or erythropoietin analogues may continue this treatment and patients may start either drug at the discretion of the investigator and according to local guidelines.

Palliative radiotherapy to specific sites is allowed if considered medically necessary by the treating physician. However the irradiated area should be as small as possible and not contain all measurable disease.

## 6.5 Other concomitant therapies

During the study, patients are not allowed to receive any other anticancer treatment including chemotherapy, immunotherapy, targeted agents, hormonal cancer therapy, radiation therapy other than described under 6.4 or experimental treatments.

All concomitant medications taken within 28 days prior to study drug treatment and during the clinical trial must be recorded on the CRF.

# 7. CLINICAL EVALUATION, LABORATORY TESTS AND FOLLOW-UP

## 7.1 Before treatment start

The basal clinical examination, blood counts and serum chemistry should be performed no more than 7 days before starting treatment. The radiological tumor and clinically measurable lesions assessments should be performed no more than 28 days before starting treatment. All investigations performed before this date must be repeated.

All of the following must be recorded:

1. Relevant medical history including oncological history, history of other disease (active or resolved), concomitant illnesses, and demographics.
2. Complete physical examination including also height, weight, BSA, ECOG PS and vital signs
3. Pre-existing adverse events information
4. Concomitant medications and concomitant treatments information
5. 12-lead ECG (to be repeated if abnormalities and/or clinically indicated)
6. Laboratory

- Haematology: WBC, ANC (bands and segments), platelets and hemoglobin counts
- Biochemistry: creatinine, urea, sodium, potassium, chlorine, calcium, alkaline phosphatase, AST (SGOT), ALT (SGPT), total bilirubin, NSE.

1. Pregnancy test (serum), if applicable
2. Disease assessment (within four weeks prior to start of treatment) by:

- CT scan of the thorax and upper and lower abdomen with contrast; total body FDG-PET imaging can be used instead of CT, if CT scan with contrast is not feasible (for example for contrast hypersensitivity) and only if targets lesions are measurable also without contrast (not in case for example of only liver target lesions);
- Brain CT or MRI scan should be performed only if initially abnormal or clinically indicated; MRI imaging can be performed instead of brain CT scan;
- Same diagnostic method used to first the target lesions, will be used to evaluate the response of the lesion; CDs of CT scan performed during the study must be available for possible central independent revision (see below 15.2).

## 7.2 During treatment

Nab-paclitaxel will be administered on days 1, 8, 15 every 28 days.

During treatment period and before each Nab-paclitaxel administration until disease progression, patient refusal or unacceptable toxicity:

1. Physical examinations including weight, BSA, ECOG PS and vital signs (only on day 1 of each cycle)
2. Laboratory
   - Haematology: WBC, ANC (bands and segments), platelets and haemoglobin counts
   - Biochemistry: creatinine, urea, sodium, potassium, chlorine, calcium, alkaline phosphatase, AST (SGOT), ALT (SGPT), total bilirubin, NSE.
3. Assessments of concomitant medications and treatments (only on day 1 of each cycle)
4. Assessments of adverse events (NCI CTCAE v. 4.03)

Radiological reassessment will be performed every 8 weeks (± 7 days), until end of treatment or progression by the different methods. Furthermore, brain CT scans will be performed only if initially abnormal or clinically indicated.

## 7.3 At the end of treatment

If patient has not progressed the disease should be assessed every 8 weeks (± 7 days), following the same procedure as during treatment, unless they have started a new anti-cancer therapy.

A clinical examination should be simultaneously performed. At the end of the study and / or at disease progression:

1. Assessment/recording best response
2. Assessment of response duration
3. Assessment of concomitant medications and treatments
4. Complete physical examination including also height, weight, BSA, ECOG PS and vital signs
5. Laboratory
   - Haematology: WBC, ANC (bands and segments), platelets and haemoglobin counts
   - Biochemistry: creatinine, urea, sodium, potassium, chlorine, calcium, alkaline phosphatase, AST (SGOT), ALT (SGPT), total bilirubin, NSE.
6. Assessments of adverse events (CTCAE v. 4.03).

## 7.4 After progression of the disease

The patients should be followed every 28 days for survival; medical history, concomitant medications and adverse events will be registered.

## 7.5 Summary table

|  | **Screening** | | **Treatment phase** | | **Post-treatment/Follow-up** | |
| --- | --- | --- | --- | --- | --- | --- |
|  | **Before treatment start (within 28 days prior therapy start)** | **Before treatment start (within 7 days prior therapy start)** | **Day 1 of each cycle** | **Day 8, 15 of each cycle** | **End of treatment**  **(At PD or after a maximum of 4 weeks)** | **After treatment (after PD, every 4 weeks)** |
| **Medical history** | X |  |  |  |  | X |
| **Physical examination (weight and height)** |  | X | X |  | X |  |
| **Informed Consent** | X |  |  |  |  |  |
| **Performance Status ECOG** |  | X | X |  | X |  |
| **Concomitant medications** |  | X | X |  |  | X |
| **Adverse Events** |  |  | X | X | X | X |
| **Therapy** |  |  | X | X |  |  |
| **Pregnancy Test** |  | X |  |  |  |  |
| **Haematology^** |  | X | X | X | X |  |
| **Serum Chemistry^^** |  | X | X | X | X |  |
| **ECG** |  | X | * | * | * |  |
| **CT scan** | X° |  | X°° |  | X |  |
| **Tumour assessment (RECIST criteria 1.1)** | X |  | X°° |  |  |  |
| **Brain CT or MRI** | # |  |  | *°° | *°° |  |

*: Only if initially abnormal or clinically indicated, #: optional

°: CT scan and/or FDG PET scan within four weeks prior to treatment start (see 7.1)

°°: After completion of every 2 cycles of treatment (every 8 weeks ± 7 days) until 6 cycles (see 5.2)

^: Haematology includes white blood cells, neutrophils, platelets and haemoglobin counts

^^: Serum chemistry includes creatinine, urea, sodium, potassium, chlorine, calcium, alkaline phosphatase, ASAT (SGOT), ALAT (SGPT), total bilirubin, NSE.

# 8. CRITERIA OF TUMOR EVALUATION

## 8.1 Objective tumor response

The objective tumor response will be used as the primary end-point in this trial. The objective response to treatment will be measured according to RECIST criteria v1.1.

Response criteria are essentially based on a set of measurable lesions identified at baseline as target lesions, and followed until disease progression.

The following paragraphs are a quick reference to the RECIST criteria.

### *8.1.1 Measurability of tumor lesions at baseline*

*8.1.1.1 Definitions*

- **Measurable disease** - the presence of at least one measurable lesion. If the measurable disease is restricted to a solitary lesion, its neoplastic nature should be confirmed by cytology/histology.
- **Measurable lesions** - lesions that can be accurately measured in at least one dimension, with longest diameter ≥ 20 mm. With spiral CT scan, lesion must be ≥ 10 mm in at least one dimension.
- **Non-measurable lesions** - all other lesions, including small lesions (longest diameter < 20 mm with conventional techniques or < 10 mm with spiral CT scan) and other non-measurable lesions. These include: bone lesions (osteoblastic or osteolytic); ascites; pleural / pericardial effusion; lymphangitis cutis / pulmonitis; abdominal masses that are not confirmed and followed by imaging techniques; and cystic lesions.

All measurements should be recorded in metric notation by use of a ruler or calipers. All baseline evaluations should be performed as closely as possible to the beginning of treatment, within four week before the beginning of the treatment.

In the present study, lesions in previously irradiated area will be considered as "non measurable” unless that site had subsequent evidence of progression before registration.

*8.1.1.2 Methods of measurements*

The same method of assessment and the same technique should be used to characterize each identified and reported lesion at baseline and during follow-up.

- ***Clinically detected lesions*** will be considered as measurable when they are superficial (e.g. skin nodules, palpable lymph nodes). For the case of skin lesions, documentation by colour photography -including a ruler to estimate the size of the lesion- is recommended.
- Lesions on ***chest X-ray*** are acceptable as measurable lesions when they are clearly defined and surrounded by aerated lung. However, CT is preferable.
- ***CT and MRI*** are the best currently available and reproducible methods to measure target lesions selected for response assessment. Conventional CT and MRI should be performed with contiguous cuts of 10 mm or less in slice thickness. Spiral CT should be performed using a 5 mm contiguous reconstruction algorithm; this specification applies to the lesions of the chest, abdomen and pelvis while head & neck lesions and those of the extremities usually require specific protocols.
- When the primary endpoint of the study is objective response evaluation, ***ultrasound (US)*** should not be used to measure tumor lesions that are clinically not easily accessible. It may be used as a possible alternative to clinical measurements of superficial palpable nodes, subcutaneous lesions and thyroid nodules. US might be useful to confirm the complete disappearance of superficial lesions usually assessed by clinical examination.
- The utilization of ***endoscopy*** and ***laparoscopy*** for objective tumor evaluation has not yet been validated. Their use in this specific context requires sophisticated equipment and a high level of expertise that may only be available in some centers. Therefore, the utilization of such techniques for objective tumor response should be restricted to validation purposes in reference centers. However, such techniques can be useful to confirm complete pathological response when biopsies are obtained.

### *8.1.2 Tumor response evaluation*

*8.1.2.1 Baseline documentation of “Target” and “Non-Target” lesions*

All measurable lesions up to a maximum of 2 lesions per organ and 5 lesions in total, representative of all involved organs, should be identified as target lesions and will be recorded and measured at baseline. Target lesions should be selected on the basis of their size (those with the longest diameter) and their suitability for accurate repetitive measurements (either by imaging techniques or clinically). A sum of the longest diameter for all target lesions will be calculated and reported as the *baseline sum longest diameter*. The baseline sum longest diameter will be used as reference by which characterize the objective tumor response.

All other lesions (or sites of disease) should be identified as non-target lesions and should also be recorded at baseline. Measurements are not required but the presence or absence of each should be noted throughout follow-up.

*8.1.2.2 Response Criteria*

8.1.2.2.1 Evaluation of target lesions

| Complete Response (CR): | Disappearance of all target lesions. Any pathological lymph nodes (whether target or non-target) must have reduction in short axis to < 10 mm |
| --- | --- |
| Partial Response (PR): | At least a 30% decrease in the sum of diameters of target lesions, taking as reference the baseline sum diameters. |
| Progressive disease (PD): | At least a 20% increase in the sum of diameters of target lesions, taking as references the smallest sum on study (this includes the baseline sum if that is the smallest on study). The appearance of one or more new lesions is also considered progression. |
| Stable Disease (SD): | Neither sufficient shrinkage to qualify for PR nor sufficient increase to qualify for PD, taking as references the smallest sum diameters while on study. |

8.1.2.2.2 Evaluation of non-target lesions

| Complete Response (CR): | Disappearance of all non-target lesions and normalization of tumour marker level. All lymph nodes must be non-pathological in size (<10 mm short axis). |
| --- | --- |
| Non-CR/Non-PD: | Persistence of one or more non-target lesion(s) and/or maintenance of tumor marker level above the normal limits. |
| Progressive disease (PD): | Appearance of one or more new lesions and/or unequivocal progression of existing non-target lesions (*). |
| (*) Although a clear progression of “non-target” lesions only is exceptional, in such circumstances, the opinion of the treating physician should prevail and the progression status should be confirmed later on by the review panel (or study chair). | |

8.1.2.2.3 Evaluation of best overall response

The best overall response is the best response recorded from the start of the treatment until disease progression/recurrence. In general, the patient's best response assignment will depend on the achievement of both measurement and confirmation criteria

| **Target lesions** | **Non-Target lesions** | **New Lesions** | **Overall Response** |
| --- | --- | --- | --- |
| CR | CR | No | **CR** |
| CR | Non-CR/Non-PD | No | **PR** |
| CR  PR | Not evaluated  Non-PD or not evaluated | No  No | **PR**  **PR** |
| SD | Non-PD or not evaluated | No | **SD** |
| Not all evaluated  PD | Non-PD  Any | No  Yes or No | **Invaluable**  **PD** |
| Any | PD | Yes or No | **PD** |
| Any | Any | Yes | **PD** |

Patients with a global deterioration of health status requiring discontinuation of treatment without objective evidence of disease progression at that time should be reported as “symptomatic deterioration”. Every effort should be made to document the objective progression even after discontinuation of treatment.

In some circumstances it may be difficult to distinguish residual disease from normal tissue. When the evaluation of complete response depends upon this determination, it is recommended the investigation of the residual lesion (fine needle aspirate/biopsy) before confirming the complete response status.

8.1.2.2.4 Frequency of tumor re-evaluation

In the present study, tumor will be re-evaluated after completion of every 2 cycles of treatment, i.e. approximately every 8 weeks ±1 week, and at least 4 weeks after the first observation of a complete or partial response. After discontinuation of protocol treatment, patients who have not progressed will still be re-evaluated every 8 weeks ±1 week, unless they have started a new anti-cancer therapy.

*8.1.2.3 Confirmatory measurements and duration of response*

8.1.2.3.1 Confirmation

The main goal of confirmation of objective response is to minimize the risk of overestimation of the response rate. In the present study, all responses need to be confirmed*.*

To be assigned a status of PR or CR, changes in tumor measurements must be confirmed by repeat assessments that should be performed no less than 4 weeks after the criteria for response are first met.

In the case of SD, follow-up measurements must have met the SD criteria at least once after study entry at a minimum interval of 8 weeks*.*

8.1.2.3.2 Duration of overall response

The duration of overall response is measured from the time measurement criteria are first met for CR/PR (whichever is first recorded) until the first date that recurrent or progressive disease is objectively documented (taking as reference for progressive disease the smallest measurements recorded since the treatment started). The duration of overall complete response is measured from the time measurement criteria are first met for CR until the first date that recurrent disease is objectively documented.

8.1.2.3.3 Duration of stable disease

Stable disease is measured from the start of the treatment until the criteria for progression are met, taking as reference the smallest sum on study (if the baseline sum is the smallest, this is the reference for calculation of PD).

### *8.1.3 Reporting of results*

All patients included in the study must be assessed for response to treatment, even if there is major protocol treatment deviation or if they are ineligible. Each patient will be assigned to one of the following categories: 1) complete response, 2) partial response, 3) stable disease, 4) progressive disease, 5) early death from malignant disease, 6) early death from toxicity, 7) early death from other cause or 9) unknown (not assessable, insufficient data). In the protocol for each patient the best overall response assessed during study treatment will be considered

In the present protocol, early death will be defined as any death occurring before the first time point of tumor re-evaluation, i.e. at the end of second chemotherapy course.

Patients for whom response is not confirmed will be classified as "unknown", unless they meet the criteria of stable disease (or the criteria of partial response in case of an unconfirmed complete response).

# 9. EVALUATION OF SAFETY

## 9.1 Monitoring, Recording and Reporting of Adverse Events

An adverse event (AE) is any noxious, unintended, or untoward medical occurrence that may appear or worsen in a subject during the course of a study. It may be a new intercurrent illness, a worsening concomitant illness, an injury, or any concomitant impairment of the subject’s health, including laboratory test values, regardless of etiology. Any worsening (i.e., any clinically significant adverse change in the frequency or intensity of a pre-existing condition) should be considered an AE. A diagnosis or syndrome should be recorded on the AE page of the CRF rather than the individual signs or symptoms of the diagnosis or syndrome. All AEs will be recorded on the CRF; the investigator will decide whether those events are suspected to be drug-related or not (see chapter below) and his decision will be recorded on the forms for all adverse events. An overdose, accidental or intentional, whether or not it is associated with an AE, or abuse, withdrawal, sensitivity or toxicity to an investigational product should be reported as an AE. If an overdose is associated with an AE, the overdose and adverse event should be reported as separate terms.

All subjects will be monitored for AEs during the study. Assessments may include monitoring of any or all of the following parameters: the subject’s clinical symptoms, laboratory, pathological, radiological or surgical findings, physical examination findings, or other appropriate tests and procedures.

All AEs will be recorded by the Investigator from the time the subject signs informed consent to 28 days post last dose of IP and every 28 days for the next 6 months, thereafter, as long-term follow-up period to collect later study-related AEs. AEs and serious adverse events (SAEs) will be recorded on the AE page of the CRF and in the subject’s source documents. All SAEs must be reported to Gruppo Oncologico Italiano di Ricerca Clinica (GOIRC) within 24 hours of the Investigator’s knowledge of the event by facsimile, or other appropriate method, using the SAE Report Form, or approved equivalent form.

Adverse events not drug related (i.e. reported as unrelated or unlikely related) will not be considered as side effects or toxicity, but reported separately.

## 9.2 General evaluation of side-effects

A qualified Investigator will evaluate all adverse events as to:

Seriousness

A serious adverse event (SAE) is any AE occurring at any dose that:

- Results in death;
- Is life-threatening (i.e., in the opinion of the Investigator, the subject is at immediate risk of death from the AE);
- Requires inpatient hospitalization or prolongation of existing hospitalization (hospitalization is defined as an inpatient admission, regardless of length of stay);
- Results in persistent or significant disability/incapacity (a substantial disruption of the subject’s ability to conduct normal life functions);
- Is a congenital anomaly/birth defect;
- Constitutes an important medical event.

Important medical events are defined as those occurrences that may not be immediately life threatening or result in death, hospitalization, or disability, but may jeopardize the subject or require medical or surgical intervention to prevent one of the other outcomes listed above. Medical and scientific judgment should be exercised in deciding whether such an AE should be considered serious.

Events **not considered** to be SAEs are hospitalizations for:

- A standard procedure for protocol therapy administration. However, hospitalization or prolonged hospitalization for a complication of therapy administration will be reported as an SAE.
- Routine treatment or monitoring of the studied indication not associated with any deterioration in condition.
- The administration of blood or platelet transfusion as routine treatment of studied indication. However, hospitalization or prolonged hospitalization for a complication of such transfusion remains a reportable SAE.
- A procedure for protocol/disease-related investigations (e.g., surgery, scans, endoscopy, sampling for laboratory tests, bone marrow sampling). However, hospitalization or prolonged hospitalization for a complication of such procedures remains a reportable SAE.
- Hospitalization or prolongation of hospitalization for technical, practical, or social reasons, in absence of an AE.
- A procedure that is planned (i.e., planned prior to starting of treatment on study) must be documented in the source document and the CRF. Hospitalization or prolonged hospitalization for a complication remains a reportable SAE.
- An elective treatment of a pre-existing condition unrelated to the studied indication.
- Emergency outpatient treatment or observation that does not result in admission, unless fulfilling other seriousness criteria above.

If an AE is considered serious, both the AE page/screen of the CRF and the SAE Report Form must be completed.

For each SAE, the Investigator will provide information on severity, start and stop dates, relationship to IP, action taken regarding IP, and outcome.

Severity / Intensity

For both AEs and SAEs, the Investigator must assess the severity / intensity of the event.

The severity / intensity of AEs will be graded based upon the subject’s symptoms according to the current active minor version of National Cancer Institute (NCI) Common Terminology Criteria for Adverse Events (CTCAE, Version 4.0); [http://ctep.cancer.gov/protocolDevelopment/electronic_applications/ctc.htm#ctc_40](http://ctep.cancer.gov/protocolDevelopment/electronic_applications/ctc.htm#ctc_40)

AEs that are not defined in the NCI CTCAE should be evaluated for severity / intensity according to the following scale:

- *Grade 1 = Mild – transient or mild discomfort; no limitation in activity; no medical intervention/therapy required*
- *Grade 2 = Moderate – mild to moderate limitation in activity, some assistance may be needed; no or minimal medical intervention/therapy required*
- *Grade 3 = Severe – marked limitation in activity, some assistance usually required; medical intervention/therapy required, hospitalization is possible*
- *Grade 4 = Life threatening – extreme limitation in activity, significant assistance required; significant medical intervention/therapy required, hospitalization or hospice care probable*
- *Grade 5 = Death - the event results in death]*

The term “severe” is often used to describe the intensity of a specific event (as in mild, moderate or severe myocardial infarction); the event itself, however, may be of relatively minor medical significance (such as severe headache). This criterion is *not* the same as “serious” which is based on subject/event *outcome* or *action* criteria associated with events that pose a threat to a subject’s life or functioning.

Seriousness, not severity, serves as a guide for defining regulatory obligations.

Causality

The Investigator must determine the relationship between the administration of IP and the occurrence of an AE/SAE as Not Suspected or Suspected as defined below:

| Not suspected: | The temporal relationship of the adverse event to IP administration makes **a causal relationship unlikely or remote**, or other medications, therapeutic interventions, or underlying conditions provide a sufficient explanation for the observed event. |
| --- | --- |
| Suspected: | The temporal relationship of the adverse event to IP administration makes **a causal relationship possible**, and other medications, therapeutic interventions, or underlying conditions do not provide a sufficient explanation for the observed event. |

Duration

For both AEs and SAEs, the Investigator will provide a record of the start and stop dates of the event.

Action Taken

The Investigator will report the action taken with IP as a result of an AE or SAE, as applicable (e.g., discontinuation or reduction of IP, as appropriate) and report if concomitant and/or additional treatments were given for the event.

Outcome

The investigator will report the outcome of the event for both AEs and SAEs.

All SAEs that have not resolved upon discontinuation of the subject’s participation in the study must be followed until recovered, recovered with sequelae, not recovered (death due to another cause) or death (due to the SAE).

## 9.3 Abnormal Laboratory Values

An abnormal laboratory value is considered to be an AE if the abnormality:

- Results in discontinuation from the study;
- Requires treatment, modification/ interruption of IP dose, or any other therapeutic intervention; or
- Is judged to be of significant clinical importance.

Regardless of severity grade, only laboratory abnormalities that fulfil a seriousness criterion need to be documented as a serious adverse event (AE).

If a laboratory abnormality is one component of a diagnosis or syndrome, the diagnosis or syndrome will be recorded on the AE page/screen of the CRF. If the abnormality is not a part of a diagnosis or syndrome, the laboratory abnormality will be recorded as the AE.

## 9.4 Pregnancy

Females of Childbearing Potential:

Pregnancies and suspected pregnancies (including a positive pregnancy test regardless of age or disease state) of a female subject occurring while the subject is on IP, or within 28 days post last dose are considered immediately reportable events. IP has to be discontinued immediately. The pregnancy, suspected pregnancy, or positive pregnancy test must be reported to the GOIRC who will inform Celgene immediately using Pregnancy Reporting Form provided by Celgene or an approved equivalent form.

The female subject should be referred to an obstetrician-gynecologist, preferably one experienced in reproductive toxicity for further evaluation and counseling.

The Investigator will follow the female subject until completion of the pregnancy, and must notify to the GOIRC immediately about the outcome of the pregnancy (either normal or abnormal outcome).

If the outcome of the pregnancy was abnormal (e.g., spontaneous or therapeutic abortion), the Investigator should report the abnormal outcome as an AE. If the abnormal outcome meets any of the serious criteria, it must be reported as a SAE within 24 hours of the Investigator’s knowledge of the event using the SAE Report Form, or approved equivalent form.

All neonatal deaths that occur within 28 days of birth should be reported, without regard to causality, as SAEs. In addition, by using the SAE Report Form (or approved equivalent form) the Investigator should report any infant death after 28 days suspected as related to the in utero exposure to the IP within 24 hours of the Investigator’s knowledge of the event.

## 9.5 Expedited Reporting of Adverse Events

### *9.5.1 Reporting to Regulatory Authorities and the Ethics Committee*

The Sponsor will inform relevant Regulatory Authorities and Ethics Committees;

- On all relevant information regarding serious unexpected adverse events suspected to be related to the IP (considering the Investigator Brochure) that are fatal or life-threatening as soon as possible, and in any case no later than seven days after knowledge of such a case. Relevant follow-up information for these cases will be subsequently be submitted within an additional eight days
- On all other serious unexpected events suspected to be related to the IP as soon as possible, but within a maximum of fifteen days of first knowledge by the investigator.

### *9.5.2 Immediate reporting by Investigator to Sponsor and Sponsor to Celgene*

The investigator will inform the Sponsor of all SAEs within 24 hours in order to allow an exact regulatory reporting obligation within the required timeframes by the Sponsor.

The Sponsor will supply appropriate MAH with a copy of all SAEs, which involve *exposure* to its product within 24 hours of being made aware of the event regardless of whether or not the event is listed in the reference document (e.g. IB, SmPC).

The Sponsor will provide the appropriate MAH with a copy of the annual periodic safety report e.g. Development Update Safety Report (DSUR) at the time of submission to the Regulatory Authority and Ethics Committee.

## 9.6 Toxic deaths

Toxic death is defined as death due to toxicity. This must be reported on the summary form. The cause of death must be reported as "toxicity".

The evaluation of toxic deaths is independent of the evaluation of response (patients can die from toxicity after a complete assessment of the response to therapy).

## 9.7 Evaluability of toxicity

All patients who have started the treatment will be included in overall toxicity analyses.

For haematological toxicity, the Study Coordinator may decide that blood counts have not been performed and/or reported according to the protocol and are therefore inadequate for the evaluation of one/several haematological parameters in those patients.

Patients who have discontinued treatment because of toxicity will always be included in the toxicity analyses.

# 10. STATISTICAL CONSIDERATIONS

## 10.1 Statistical design

### *10.1.1 Sample size*

The aim of this study is to decide if Nab-paclitaxel tumor response rate (the percentage of partial and complete responses) at the end of a maximum of 6 courses in either of the two groups, *sensitive* or *refractory* relapsed SCLC, is sufficient to justify further investigation of the drug in these patients’ populations.

*Refractory* and *sensitive* disease is defined according to treatment free interval (TFI), i.e. the interval from the last chemotherapy administration during first-line therapy and the occurrence of progressive disease. Disease is considered to be *refractory* if TFI is < 60 days, while it is considered to be *sensitive* if TFI is > 60 days.

*Refractory disease*

A response rate ≤ 5% would be considered of no further interest. According to the Fleming’s single stage design, assuming that the experimental treatment could guarantee a response rate ≥ 20%, for a 5% significance level and 80% power, 22 patients with refractory disease are needed to be enrolled into the study. If 4 or more objective responses will be observed it can be concluded that a response rate > 5% is possible.

*Sensitive disease*

A response rate ≤ 15% would be considered of no further interest. According to the Fleming’s single stage design, assuming that the experimental treatment could guarantee a response rate ≥ 30%, for a 5% significance level and 80% power, 43 patients with sensitive disease are needed to be enrolled into the study. If 11 or more objective responses will be observed it can be concluded that a response rate >15% is possible.

The study is not designed to perform any comparison between the 2 cohorts.

### *10.1.2 Stratifications*

Patients will be classified according to treatment free interval (TFI), i.e. the interval from the last chemotherapy administration during first-line therapy and the occurrence of progressive disease. Disease is considered to be *refractory* if TFI is < 60 day, while it is considered to be *sensitive* if TFI is ≥ 60 days.

## 10.2 Statistical analysis plan

### *10.2.1 Primary and secondary endpoints*

The primary end-point is objective tumor response that will be evaluated according to standard RECIST 1.1 criteria and will be based on the Investigator’s assessment. Patients with no tumor assessment after baseline will be classified as non-responders.

Secondary objectives include safety, Progression Free Survival (PFS) and Overall survival (OS)

**Safety evaluation**: the assessment of safety will be based mainly on the frequency of adverse events. Adverse events will be summarized by presenting the number and percentage of patients having any adverse event, having an adverse event in each body system and having each individual adverse event.

**PFS** will be calculated from the patient registration to the evidence of progressive disease, or death, or the last date the patient was known to be progression-free or alive.

**OS** will be calculated from the registration to death from any cause, or the last date the patient was known to be alive.

Probabilities of progression‑free survival and probabilities of overall survival will be estimated according to the Kaplan and Meier product‑limit method.

### *10.2.2 Analysis populations’*

*Registered population*

Registered population will include all patients who were enrolled into the trial.

*Modified intention to treat population*

Modified intention to treat population will include all patients who were enrolled into the trial and received at least one dose of the study drug.

The assignment of patients to the study populations will be performed before the database lock.

### *10.2.3 Statistical methods*

For the primary endpoint and all secondary endpoints, the modified intention to treat population will be analysed. Descriptive tables will be produced for the tumor response rate and the best overall response. Exact binomial method will be used to estimate the response rate (CR+PR) and its 95% confidence interval.

Probabilities of progression‑free survival and probabilities of overall survival will be estimated according to the Kaplan and Meier product‑limit method.

Toxicity descriptive tables will be produced which provide the worst degree of toxicity measured over all cycles according to the CTCAE version 4.03.

Separate analyses will be carried out in the two groups of sensitive relapse disease patients and refractory relapse disease ones.

As this is a phase II trial, no statistical comparisons will be made between the groups and no p values will be provided.

### *10.2.4 Prognostic factor analyses*

Separate analyses will be made in groups of sensitive relapse disease patients and refractory relapse disease ones, however no prognostic factor analyses are foreseen.

### *10.2.5 Data recording and display*

Frequency tables will be tabulated for all categorical variables by the levels of the variables as they appear on the CRF (with percentages). Categories with a text field specification will be tabulated as categories and then supplemented by a listing with the following information for the patients fulfilling the condition for the specification (patient id, institution, value of the item and text field contents).

Dates relating to events after entry will be presented as the delay in days (or weeks, months, or years) between the event and the date of entry (date of event - date of registration + 1) and presented using the median and range. For example, the date of last administration of treatment will be presented as the time elapsed (in days, weeks, months or years, as appropriate) between the day of the last administration and the date of entry on study (date of last administration – date of registration +1).

Dates relating to events prior to entry will be presented as the delay in days (or weeks, months, or years) between the past event and the date of entry (date of registration – date of past event + 1) and presented using the median and range. For example, on the registration checklist, the date of last administration of prior treatment (or the date of first diagnosis of the cancer) will be presented as the time elapsed (in days, weeks, months or years, as appropriate) since the day of the last administration and the date of entry on study (date of registration – last administration/diagnosis +1).

Other delays (e.g. re-treatment delays) are presented as continuous variables using the median and range.

Continuous variables for which a coding system exists (such as for laboratory data) will be recoded into categories (for adverse events, the grading scale according to the CTCAE version 4.03 will be used). Whenever no specific scale exists, lab data will be categorized based on the normal range: for example, below the lower normal limit (when appropriate), within the normal range, above the upper limit for normal (ULN) and the degree to which it is above the ULN (for example > 2.5 x ULN, > 5 x ULN, > 10 x ULN). For laboratory data, the nadir is generally displayed. The nadir in a given cycle is the lowest laboratory value in that cycle; the overall nadir for a patient is the lowest laboratory value among all cycles.

Other continuous variables (for example age, dose) are presented using the median and range (minimum, maximum).

Continuous data may also be presented in categories (for example, age may also be grouped in decades).

## 10.3 Interim analyses

No interim analyses are foreseen in this study.

## 10.4 End of study

End of study occurs when all of the following criteria have been satisfied:

1. Thirty days after all patients have stopped protocol treatment
2. The trial is mature for the analysis of the primary endpoint as defined in the protocol;
3. The database has been fully cleaned and frozen for this analysis.

# 11. DATA MONITORING

A Data and Safety Monitoring Board (DSMB) will monitor the recruitment, the reported adverse events and the data quality at least twice a year. Arising problems will be discussed with the Study Coordinator who will take appropriate measures. Relevant information (including relevant safety data) will be included in the study status reports. No efficacy results will be presented at investigators meetings before the trial is closed to recruitment and data are mature for the analysis of the primary endpoint, unless recommended otherwise by the (DSMB).

# 12. INVESTIGATOR AUTHORIZATION PROCEDURE

Investigators will be authorized to register patients in this trial only when they have returned to the Data Center:

- The updated signed and dated Curriculum Vitae of the Principle Investigator.
- The signature log-list of the staff members with a sample of each authorized signature and the indication of the level or delegations.
- The coordinates of the pharmacist who will be responsible for the trial medication.

The new investigator will be added to the “authorization list”, and will be allowed to register patients in the trial as soon as:

- All the above-mentioned documents are available at the Data Center.
- All applicable national legal and regulatory requirements are being fulfilled.

# 13. PATIENT REGISTRATION PROCEDURE

This is a multicenter randomized phase II trial. All patients must be registered by the Data Center prior to start of treatment. Patient registration will only be accepted from authorized investigators (see section 12), who will receive a personal user ID to access the registration system.

A patient who has not been registered before the first treatment administration will not be accepted for the study at a later date.

Randomization will be centralized and performed online connecting to the following URL: <https://www.eclintrials.org/ect/>

This must be done before the start of the protocol treatment.

At the end of the procedure, a patient sequential identification number and an arm of treatment will be allocated to the patients. The sequential identification number attributed to the patient at the end of the registration procedure identifies the patient.

# 14. FORMS AND PROCEDURES FOR COLLECTING DATA

Case report forms and schedule for completion will be available on-line. Data will be reported on the forms at the following URL: <https://www.eclintrials.org/ect/>

The case report forms (CRFs) must be completed, dated and signed by the investigator or one of his/her authorized staff members as soon as the requested information is available. The list of staff members authorized to sign case report forms (with a sample of their signature) must be sent to the Data Center by the responsible investigators before the start of the study.

The Data Center will perform extensive consistency checks on the CRFs and issue Query Forms in case of inconsistent data. Those Query Forms must be immediately answered and signed by the investigator (or an authorized staff member).

# 15. QUALITY ASSURANCE

## 15.1 Control of data consistency

Computerized and manual consistency checks will be performed on newly entered forms; queries will be issued in case of inconsistencies. Consistent forms will be validated by the Data Center to be entered on the master database. Inconsistent forms will be kept "pending" until resolution of the inconsistencies.

## 15.2 Central review procedures

To ensure consistency of tumor response measurements among centers, investigator determined responses may be reviewed by an independent panel of oncologists and radiologists. CT films, radiograms and medical records of the patients enrolled in this trial should be retained at each center until notified by the sponsor and should be made available for review upon request.

# 16. ETHICAL CONSIDERATIONS

## 16.1 Patient protection

The responsible investigator will ensure that this study is conducted in agreement with the Declaration of Helsinki. The protocol has been written, and the study will be conducted according to the ICH Harmonized Tripartite Guideline for Good Clinical Practice. The protocol will be approved by the Local Ethics Committees.

## 16.2 Subject identification

The name of the patient will not be asked for nor recorded at the Data Center. A sequential identification number will be automatically attributed to each patient registered in the trial. This number will identify the patient and must be included on all case report forms. In order to avoid identification errors, patient’s code (maximum of 4 letters), date of birth and local chart number (if available) will also be reported on the case report forms.

## 16.3 Informed consent

All patients will be informed of the aims of the study, the possible adverse events, the procedures and possible hazards to which he/she will be exposed, and the mechanism of treatment allocation.

They will be informed as to the strict confidentiality of their patient data, but that their medical records may be reviewed for trial purposes by authorized individuals other than their treating physician.

The informed consent form is part of the documents to be submitted to the ethics committee for approval. The competent ethics committee for each institution must validate local informed consent documents before the center can join the study. It is the responsibility of the Local Ethical Committee to guarantee that the translation is conforming to the ICH-GCP guidelines.

It will be emphasized that the participation is voluntary and that the patient is allowed to refuse further participation in the protocol whenever he/she wants. This will not prejudice the patient’s subsequent care. Documented informed consent must be obtained for all patients included in the study before they are registered or randomized at the Data Center. This must be done in accordance with the national and local regulatory requirements.

For European Union member states, the informed consent procedure must conform to the ICH guidelines on Good Clinical Practice. This implies that “the written informed consent form should be signed and personally dated by the patient or by the patient’s legally acceptable representative”.

# 17. ADMINISTRATIVE RESPONSABILITIES

## 17.1 The study coordinators

The Study Coordinators (in cooperation with the Data Center) will be responsible for writing the protocol, reviewing all case report forms and documenting his/her review on evaluation forms, discussing the contents of the reports with the Data Center and for publishing the study results. The Study Coordinators will also generally be responsible for answering all clinical questions concerning eligibility, treatment, and the evaluation of the patients.

**Principal Investigator**

*Dr. Andrea Ardizzoni*

Director of Medical Oncology Unit,

Dept. Oncology-Haematology,

S. Orsola-Malpighi University Hospital

Via Albertoni 15, 40138 Bologna, Italy

Phone/Fax:+39051636220/+390516362207

E-mail: [andrea.ardizzoni@aosp.bo.it](mailto:andrea.ardizzoni@aosp.bo.it)

**Scientific Study Coordinator**

*Dr. Marcello Tiseo*

Medical Oncology Unit

University Hospital of Parma

Via Gramsci 14, 43126, Parma, Italy

Phone/Fax: +390521702316/+390521995448

e-mail: [mtiseo@ao.pr.it](mailto:mtiseo@ao.pr.it)

## 17.2 Clinical Trial Office and Data Center

The Data Center will be responsible for reviewing the protocol, collecting case report forms, controlling the quality of the reported data, and generating reports and analyses in cooperation with the Study Coordinator. All methodological questions should be addressed to the Data Center.

**Clinical Trial Office**

*Dr. Michele Tognetto*

Medical Oncology Unit,

Dept. Oncology-Haematology,

S. Orsola-Malpighi University Hospital

Via Albertoni 15, 40138 Bologna, Italy

Phone/Fax: +390512142204/+390516362508

E-mail: [nabsterstudy@gmail.com](mailto:nabsterstudy@gmail.com)

***Data Center***

*Dr. Luca Boni*

Centro per il Coordinamento delle Sperimentazioni Cliniche

Istituto Toscano Tumori

c/o AOU Careggi - Padiglione 17 San Damiano

L.go Brambilla, 3 – 50134 Firenze

Phone/Fax:+390557945490/+390557947553

E-mail: [luca.boni@ittumori.it](mailto:luca.boni@ittumori.it)

**Safety Desk:**

Themas srl

Cassina Plaza

Via Roma 108 – Edificio F, Scala 2

20060 Cassina De’ Pecchi (MI)

The Safety Desk will forward all SAE within 24 hours of receipt to , the Study Coordinators and the Data Center.

All unexpected SADR will additionally be forwarded to all participating investigators.

The Safety Desk will take in charge the regulatory reporting to the National Authorities in cooperation with the Regulatory Desk Manager whenever applicable.

The Safety Desk will provide a six-monthly summary of all SAE reports which will be added in the group meeting report to which will be distributed to all participating investigators.

# 18. TRIAL SPONSORSHIP

This study is sponsored by GOIRC (Gruppo Oncologico Italiano di Ricerca Clinica). The protocol will be supported by grant from Celgene.

# 19. TRIAL INSURANCE

An insurance certificate will be made available to the participating sites at the time of study initiation.

Clinical trial insurance is only valid if the treatment is given in a center authorized by the Data Center and which has obtained Ethical Committee approval (individually or centrally depending on the national regulations applicable).

# 20. PUBLICATION POLICY

The final publication of the trial results will be written by the Study Coordinators on the basis of the final analysis performed at the Data Center. After revision by the Data Center and other co-authors the manuscript will be sent to a major scientific journal. Authors of the manuscript will include at least the Study Coordinators, the members of the writing committee, the investigators who have included more than 10% of the eligible patients in the trial (by order of inclusion).

The manuscript will include an appropriate acknowledgment section, mentioning all investigators who have contributed to the trial, the data center staff involved in the study, as well as supporting bodies.

All publications including data from the present trial will be submitted for review to the Data Center and to all co-authors prior to submission. The Study Coordinators and the Data Center must approve all publications, abstracts and presentations of data pertaining to patients included in this study.

# 21. REFERENCES

1. Ardizzoni, A., Tiseo, M. & Boni, L. Validation of standard definition of sensitive versus refractory relapsed small cell lung cancer: A pooled analysis of topotecan second-line trials. *Eur. J. Cancer* 50, 2211–2218 (2014).

2. O’Brien, M. E. R. *et al.* Phase III trial comparing supportive care alone with supportive care with oral topotecan in patients with relapsed small-cell lung cancer. *J. Clin. Oncol.* 24, 5441–5447 (2006).

3. Von Pawel, J. *et al.* Topotecan Versus Cyclophosphamide, Doxorubicin, and Vincristine for the Treatment of Recurrent Small-Cell Lung Cancer. *J. Clin. Oncol.* 17, 658–67 (1999).

4. Ardizzoni, A. Topotecan in the Treatment of Recurrent Small Cell Lung Cancer: An Update. *Oncologist* 9, 4–13 (2004).

5. Smit, E. F. *et al.* A phase II study of paclitaxel in heavily pretreated patients with small-cell lung cancer. *Br. J. Cancer* 77, 347–351 (1998).

6. Groen, H. *et al.* Paclitaxel and carboplatin in the treatment of small-cell lung cancer patients resistant to cyclophosphamide, doxorubicin, and etoposide: a non-cross-resistant schedule. *J Clin Oncol.* 17, 927–32 (1999).

7. De Jong, W. K. *et al.* Phase III study of cyclophosphamide, doxorubicin, and etoposide compared with carboplatin and paclitaxel in patients with extensive disease small-cell lung cancer. *Eur. J. Cancer* 43, 2345–2350 (2007).

8. Mori, K., Kamiyama, Y., Kondo, T., Kano, Y. & Kodama, T. Phase II study of weekly chemotherapy with paclitaxel and gemcitabine as second-line treatment for advanced non-small cell lung cancer after treatment with platinum-based chemotherapy. *Cancer Chemother. Pharmacol.* 60, 189–95 (2007).

9. Socinski, M. a *et al.* A dose finding study of weekly and every-3-week nab-Paclitaxel followed by carboplatin as first-line therapy in patients with advanced non-small cell lung cancer. *J. Thorac. Oncol.* 5, 852–861 (2010).

10. Socinski, M. A. *et al.* Weekly nab-paclitaxel in combination with carboplatin versus solvent-based paclitaxel plus carboplatin as first-line therapy in patients with advanced non-small-cell lung cancer: final results of a phase III trial. *J. Clin. Oncol.* 30, 2055–62 (2012).

11. Socinski, M. a. *et al.* Safety and efficacy of weekly nab®-paclitaxel in combination with carboplatin as first-line therapy in elderly patients with advanced non-small-cell lung cancer. *Ann. Oncol.* 24, 314–321 (2013).

12. Gelderblom, H., Verweij, J., Nooter, K. & Sparreboom, A. Cremophor EL. *Eur. J. Cancer* 37, 1590–1598 (2001).

13. Yardley, D. A. nab-Paclitaxel mechanisms of action and delivery. *J. Control. Release* 170, 365–72 (2013).

14. Riondel, J. *et al.* Therapeutic response to taxol of six human tumors xenografted into nude mice. *Cancer Chemother. Pharmacol.* 17, 137–42 (1986).

15. Desai, N. *et al.* Increased antitumor activity, intratumor paclitaxel concentrations, and endothelial cell transport of cremophor-free, albumin-bound paclitaxel, ABI-007, compared with cremophor-based paclitaxel. *Clin. Cancer Res.* 12, 1317–24 (2006).

16. Ibrahim, N. K. *et al.* Phase I and Pharmacokinetic Study of ABI-007, a Cremophor-free, Protein-stabilized, Nanoparticle Formulation of Paclitaxel. *Clin. Cancer Res.* 8, 1038–1044 (2002).

17. Nyman, D. W. *et al.* Phase I and pharmacokinetics trial of ABI-007, a novel nanoparticle formulation of paclitaxel in patients with advanced nonhematologic malignancies. *J. Clin. Oncol.* 23, 7785–93 (2005).

18. Gardner, E. R. *et al.* Randomized crossover pharmacokinetic study of solvent-based paclitaxel and nab-paclitaxel. *Clin. Cancer Res.* 14, 4200–5 (2008).

19. Sparreboom, A. *et al.* Comparative preclinical and clinical pharmacokinetics of a cremophor-free, nanoparticle albumin-bound paclitaxel (ABI-007) and paclitaxel formulated in Cremophor (Taxol). *Clin. Cancer Res.* 11, 4136–43 (2005).

20. Huizing, M. T. *et al.* Pharmacokinetics of paclitaxel and three major metabolites in patients with advanced breast carcinoma refractory to anthracycline therapy treated with a 3-hour paclitaxel infusion: A European Cancer Centre (ECC) trial. *Ann. Onc.* 6, 699–704 (1995).

21. Organ Dysfunction Working Group, N. C. I. ctep.cancer.gov/protocoldevelopment/docs/hepatic_dysfunction_v3.doc.

22. Gradishar, W. J. *et al.* Phase III trial of nanoparticle albumin-bound paclitaxel compared with polyethylated castor oil-based paclitaxel in women with breast cancer. *J. Clin. Oncol.* 23, 7794–803 (2005).

23. Von Hoff, D. D. *et al.* Increased survival in pancreatic cancer with nab-paclitaxel plus gemcitabine. *N. Engl. J. Med.* 369, 1691–703 (2013).

24. Grilley-Olson, J. E. *et al.* A randomized phase II study of carboplatin with weekly or every-3-week nanoparticle albumin-bound paclitaxel (abraxane) in patients with extensive-stage small cell lung cancer. *Oncologist* 20, 105–6 (2015).

**S2.**

| **Center** | **P.I** |
| --- | --- |
| U.O.C. Oncologia Medica, Azienda Ospedaliero-Universitaria di Parma, Parma, Italy | prof. Marcello Tiseo |
| U.O. Oncologia Medica, Ospedale Versilia, Lido di Camaiore, Italy | dott. Andrea Camerini |
| U.O.C. Oncologia Medica, Azienda Ospedaliera S. Croce e Carle, Cuneo, Italy | dott. Ida Colantonio |
| U.O.C. Oncologia Medica, ASST Cremona, Cremona, Italy | dott. Matteo Brighenti |
| U.O.C. Oncologia Medica, Azienda Ospedaliero-Universitaria Policlinico di Modena, Modena, Italy | dott. Fausto Barbieri |
| U.O.C. Oncologia Medica, ASST Papa Giovanni XXIII, Bergamo, Italy | dott. Anna Cecilia Bettini |
| U.O.C. Oncologia Medica, IRST di Meldola, Meldola, Italy | dott. Angelo Delmonte |
| U.O.C. Oncologia Medica, Casa Sollievo della Sofferenza, San Giovanni Rotondo, Italy | dott. Vito D’Alessandro |
| U.O.C. Oncologia Medica, AUSL della Romagna, Ospedale di Ravenna, Ravenna, Italy | dott. Claudio Dazzi |
| U.O. Oncologia Medica, AUSL di Modena, Ospedale di Carpi, Carpi, Italy | dott. Lucia Longo |
| U.O. Oncologia Medica, Azienda Toscana Nord Ovest, Ospedale di Lucca, Lucca, Italy | dott. Carmelo Tibaldi |
| U.O.C. Oncologia Medica, Ospedale Sacro Cuore Don Calabria, Negrar, Italy | dott. Stefania Gori |
| U.O.C. Oncologia Medica, Ospedale di Summa-Perrino, Brindisi, Italy | dott. Saverio Cinieri |
| U.O.C. Oncologia Medica, Azienda Sanitaria Dell’Alto Adige, Ospedale di Bolzano, Bolzano, Italy | dott. Emanuela Vattemi |
| U.O.C. Oncologia Medica, Azienda Ospedaliera di Rilievo Nazionale-Ospedale Cardarelli, Napoli, Italy | dott. Ferdinando Riccardi |
| U.O.C. Oncologia Medica, Azienda USL di Piacenza, Piacenza, Italy | dott. Luigi Cavanna |
| U.O.C. Oncologia Medica, Azienda Ospedaliero-Universitaria di Ferrara, Ferrara, Italy | prof. Antonio Frassoldati |
| U.O.C. Oncologia Medica, Ospedale Civile di Pordenone, Pordenone, Italy | dott. Alessandro Del Conte |
| U.O.C. Oncologia Medica, ULSS9 Ospedale di Legnago, Legnago, Italy | dott. Andrea Bonetti |
| U.O.C. Oncologia Medica, Azienda Ospedaliero-Universitaria Policlinico S.Orsola-Malpighi di Bologna, Bologna, Italy | prof. Andrea Ardizzoni |
| U.O.C. Oncologia Medica, Azienda USL Ospedale di Imola, Imola, Italy | dott. Antonio Maestri |
| U.O.C. Oncologia Medica, Azienda Ospedaliera SS. Antonio e Biagio e Cesare Arrigo di Alessandria, Alessandria, Italy | dott. Pier Luigi Piovano |

**S3.**

|  | Refractory (n = 25) | Sensitive (n = 43) |
| --- | --- | --- |
| N. of courses (total) | 62 | 129 |
| N. of courses per patient (mean, SD) | 2.48  (± 1.61) | 3.00  (± 1.70) |
| N. of courses (per patients) |  |  |
| 1 | 7 (28.0%) | 4 (9.3%) |
| 2 | 11 (44.0%) | 22 (51.2%) |
| 3 | 1 (4.0%) | 3 (7.0%) |
| 4 | 3 (12.0%) | 6 (13.9%) |
| 5 | 0 | 2 (4.7%) |
| 6 | 3 (12.0%) | 5 (11.6%) |
| 8 | 0 | 1 (2.3%) |
| Dose reduction |  |  |
| No | 10 (40.0%) | 19 (44.2%) |
| Yes | 15 (60.0%) | 24 (55.8%) |
| Treatment delay |  |  |
| No | 13 (52.0%) | 22 (51.2%) |
| Yes | 12 (48.0%) | 21 (48.8%) |
| Relative dose intensity  (mean, SD) | 0.76  (± 0.21) | 0.80  (± 0.18) |
| SD, standard deviation | | |

**S4.**

| **Code** | **Name** |
| --- | --- |
| CE150081 | COMITATO ETICO INDIPENDENTE ISTITUTO CLINICO HUMANITAS |
| CE150123 | COMITATO ETICO INTERAZIENDALE ASO S.CROCE ECARLE E AA.SS.LL CN1,CN 2 E AT |
| CE150131 | COMITATO ETICO PROVINCIALE DI VARESE |
| CE150155 | COMITATO ETICO AREA CREMONA MANTOVA LODI |
| CE150046 | COMITATO ETICO PER LA SPERIMENTAZIONE CLINICA DELLE PROVINCIE DI VERONA E ROVIGO |
| CE150034 | COMITATO ETICO REGIONE TOSCANA - AREA VASTA NORD OVEST |
| CE150059 | COMITATO ETICO DELL' A.USL DI PIACENZA |
| CE150100 | COMITATO ETICO INTERAZIENDALE BOLOGNA-IMOLA |
| CE150071 | COMITATO ETICO REGIONE TOSCANA - AREA VASTA CENTRO |
| CE150114 | COMITATO ETICO PROVINCIALE DI MODENA |
| CE150044 | COMITATO INDIPENDENTE DI ETICA MEDICA ASL BR |
| CE150095 | COMITATO ETICO DELLA PROVINCIA MONZA BRIANZA |
| CE150115 | COMITATO ETICO INTERAZIENDALE AOU CITTA' DELLA SALUTE E DELLA SCIENZA DI TORINO |
| CE150102 | COMITATO ETICO IRST IRCCS E AVR |
| CE150103 | COMITATO ETICO UNICO DELLA PROVINCIA DI FERRARA |
| CE150037 | SEZ DEL CE IRCCS IST TUMORI G PAOLO II BA C/O FONDAZIONE CASA SOLLIEVO DELLA SOFFERENZA SG ROTONDO |
| CE150040 | CARDARELLI-SANTOBONO |
| CE150105 | COMITATO ETICO INTERAZIENDALE AZIENDA OSPEDALIERA "SS.ANTONIO E BIAGIO E C.ARRIGO" DI ALESSANDRIA |
| CE150118 | COMITATO ETICO DELLA PROVINCIA DI BERGAMO |
| CE150099 | COMITATO ETICO DELL'AZIENDA SANITARIA DELLA PROVINCIA AUTONOMA DI BOLZANO |
| CE150097 | COMITATO ETICO INDIPENDENTE DELL'AZIENDA OSPEDALIERO-UNIVERSITARIA DI BOLOGNA |
| CE150117 | COMITATO ETICO PER PARMA |
